# Extended reality interventions for health and procedural anxiety: An overview of reviews

**DOI:** 10.1177/20552076251411512

**Published:** 2026-02-11

**Authors:** Tom Arthur, Sophie Robinson, David Harris, Mark Wilson, Samuel Vine, GJ Melendez-Torres

**Affiliations:** Department of Public Health and Sports Sciences, Faculty of Health and Life Sciences, 3286 University of Exeter, Exeter, UK

**Keywords:** Virtual reality, fear, patient anxiety, phobia, immersive

## Abstract

**Background:**

While Extended Reality (XR) technologies are becoming increasingly prevalent across society, there is a lack of consensus around their utilisation for the management of health and medical procedure anxieties. We undertook an overview of reviews to examine the effectiveness of these technology-based interventions.

**Methods:**

Data were extracted from full-text systematic reviews of patient-directed XR interventions for health and procedural anxiety. Records from the beginning of 2013 until 30 May 2023 were obtained from searches of MEDLINE, Embase, APA PsycINFO and Epistemonikos. Narrative synthesis then examined the consistency, quality and range of eligible research evidence, and reviews were appraised using the AMSTAR-2 tool.

**Results:**

We examined 56 reviews from diverse clinical contexts (35 of which included meta-analysis). Procedural anxieties were most commonly researched, including those relating to needle insertion, acute surgery, dental operations and/or wound care. Other studies focused on more general health anxieties, relating to longer-term treatment and rehabilitation, maternity and chronic conditions. A range of interventions (e.g. distraction- and exposure-based approaches) and technologies (e.g. immersive and non-immersive devices) have been evaluated, although comparisons between different types of interventions are lacking. While XR interventions were generally found to reduce patient anxiety, AMSTAR-2 evaluations highlighted 44/46 of the appraised reviews as low or critically low in quality, and intervention reporting was often lacking in detail.

**Conclusions:**

Evidence in support of XR interventions has not reached maturity and is currently lacking. Therefore, the emerging positive consensus for these techniques should be challenged, and the rationale for adopting such techniques in practice further considered.

## Introduction

Anxiety about receiving medical treatments can be a common and debilitating experience. Around half of patients experience anxiety before a dental or surgical procedure,^1–3^ and the prevalence of health anxieties seems to be rising.^4^ While ranging from momentary worry and negative feelings to long-term phobias or conditions, these experiences can have deleterious impacts on clinical care pathways. Indeed, they can relate to both *procedural anxieties* (i.e. acute and excessive fear of a specific medical procedure^5^) and *general health anxieties* (i.e. distress about the implications of a medical condition^6^); representing challenges that are event-specific and transitory, or chronic and enduring. Classifications of procedural and general health anxieties are typically pragmatic distinctions that serve for organisational purposes, as they do in this paper hereafter. From a conceptual perspective, these distinctions may highlight diverging contextual and neuropsychological mechanisms (e.g. different situational responses or ‘triggers’), yet their practical impacts on treatment provision remain broadly similar. For instance, when unmanaged, they can form a major barrier to accessing healthcare across a range of conditions and procedures, and the avoidance of anxiety-inducing experiences can simultaneously worsen patient wellbeing and exacerbate service demands (by increasing usage^7^ and costs^8,9^). As with general treatment and patient non-attendance data,^10^ negative anxiety-related outcomes appear more common in traditionally under-served groups (e.g. those with limited support structures,^11^ less extensive education backgrounds,^12^ or from a lower socioeconomic and/or ethnic minority status^12,13^) and likely contribute to wider system inequalities. Current solutions to this problem, such as therapy and sedation, are often expensive, time-consuming and hard to access. Therefore, new and effective methods for reducing health and procedural anxiety require exploration.

Extended Reality (XR) provides a potential way of managing anxiety within healthcare settings. Here, XR refers to technologies (including augmented reality [AR], mixed reality [MR] and virtual reality [VR]) that alter a person's sensory experience through computer-generated digital assets (see Box 1). Though XR therapies for specific anxieties and phobias have existed since the 1990s, early systems were costly and technically limited. However, over the last 10–15 years, recent advancements in technological capability and availability have led to rapidly increasing adoption and implementation across health services. During this time, studies have shown that XR interventions can successfully alleviate negative anxiety-related phobias,^14,15^ stressors (e.g.^16^) and generalised conditions.^17^ Thus, given their increasing availability and affordability within practice (see recent cost evaluation analysis^18^), as well as their unique pathways for reducing service barriers and inequalities (see^19^), XR technologies represent a promising tool for improving access to healthcare.

**Table:** 

**Box 1. Extended Reality (XR) Interventions**
The term ‘XR’ describes a family of technologies that alter a person’s sensory experience by adding digital assets to physical or real-world environments. These include *Virtual Reality* (where users see and interact within a fully computer-generated environment), *Mixed Reality* (where users see computer-generated images but interact with real-world objects) and *Augmented Reality* (where users see and interact with real-world objects that are enhanced with computer-generated images). For the management of health and procedural anxieties, these technologies can be used to target various benefit pathways:
**Distraction –** XR is used to direct attention away from anxiety cues, towards more enjoyable or relaxing sensory stimuli (e.g. using games, video content, natural scenery). **Exposure –** XR is used to simulate an upcoming anxiety encounter and to gradually habituate a patient with anxiety-related cues. **Education –** XR is used to inform patients about relevant health conditions, procedures, or treatments, in an attempt to lower their anxiety about these elements. **Other –** XR can also be used for indication-specific purposes, for example, as a physical therapy tool, an ‘exergame’ platform, or for presenting 3D scan images.

Several mechanisms are implicated in XR-based patient anxiety interventions, usually classed as exposure, education and distraction. For example, XR can deliver an immersive ‘preview’ of what a medical operation will be like, as a way of educating a patient or gradually exposing them to anxiety-related cues. These interventions align with neuro-psychological processes outlined in established fear extinction and cognitive behavioural theories, such as the development of inhibitory learning and conditioning responses or the restructuring of maladaptive thoughts and beliefs. Alternatively, XR can be employed as a form of relaxation or ‘entertainment’ during medical procedures, to provide patients with more enjoyable sensory stimuli^20^ that divert attentional resources and activate more adaptive affective states (e.g. in line with information/emotional processing models).

Despite their theoretical promise, there is a lack of consensus around the application and efficacy of XR interventions for the management of patient anxiety. This is both true for general health anxieties and procedural anxieties: indeed, most research focuses on clinical contexts that lack generalisability, and conclusions are often limited to a small number of heterogeneous studies within these domains. Relatedly, interventions are consigned to specific distraction-, exposure- or education-based mechanisms, and there are few comparisons between these benefit pathways. Moreover, study interventions are seldom underpinned by any shared theoretical framework or technological standards, meaning that diverse hardware types and programmes are used with questionable commensurability. For instance, devices that provide fully immersive functionalities and naturalistic multi-sensory experiences (e.g. modern XR headsets with six degrees of freedom tracking capabilities) are often evaluated in the same category as systems that offer limited interactivity or immersive features (e.g. 360° video environments), with little consideration of how these technological capabilities may impact on patient- or programme-level outcomes. Taken together, these limitations create a fragmented and incoherent basis for clinical application, and the broad effectiveness of XR-based interventions for health and procedural anxieties remains unclear.

We undertook an overview of reviews addressing XR interventions for health and procedural anxiety to determine the strength and quality of existing review-level evidence across diverse clinical contexts. This ‘umbrella review’ approach represents the first of its kind in the topic of health and procedural anxiety, and included comparative evaluations of effectiveness, weight of evidence and applicability between different indications and interventions. Drawing on the distinctive features of our set of included studies, we also explored the types of intervention most linked with effectiveness, the impacts of XR system features, any differential patterns of effectiveness between populations, and the forms of anxiety that are most receptive to change (e.g. in terms of procedure-specific or general health anxieties).

## Methods

### Study design

An umbrella review methodology was employed, which synthesised existing systematic reviews relating to XR-based interventions for health and procedural anxiety. A protocol for the study was pre-registered on the Open Science Framework (at https://osf.io/nhzf8/), which specified the search strategy, eligibility criteria, and methods of data extraction and synthesis. Where relevant, we followed Preferred Reporting Items for Systematic Reviews and Meta-Analysis (PRISMA) guidelines,^21^ as well as the Cochrane Handbook for systematic reviews and the Preferred Reporting Items for Overviews of Reviews (PRIOR) statement.^22^

### Eligibility criteria

The review defined eligibility based on four inclusion criteria. First, included studies were restricted to systematic reviews only, as defined by standards developed for the Database of Abstracts of Reviews of Effects. Specifically, studies were classed as a systematic review based on: (1) transparent reporting of inclusion and exclusion criteria, (2) adequate searches of the literature and (3) attempted synthesis of extracted data. An ‘adequate search’ was defined from reviews that included an electronic database and a structured search query. However, reviews did not need to focus on the effectiveness of XR interventions and could be inclusive of patient experience, implementation, economic evaluation, or other related outcomes.

Our second inclusion criterion required that reviews placed sufficient focus on XR interventions. Here, a broad conceptualisation of XR was employed to capture a variety of VR-, MR- and AR-based systems. A technology was considered to be XR if it altered a person's sensory experience by adding immersive, three-dimensional digital assets to a physical or simulated environment. Thus, reviews were excluded if they did not include meaningful immersive components (e.g. evaluations of mobile applications or tablet-based devices) or if XR was not synthesised as a discrete category or subgroup in their analyses.

Third, we included reviews of interventions that are patient-directed for health and procedural anxiety. By ‘patient-directed’, we refer to interventions that are designed to be used directly by patients, as opposed to those, for example, intended for training clinicians. No restrictions were made in relation to the specific patient population involved in a study. We included reviews of interventions that addressed either general health anxieties or specific anxieties about medical procedures (e.g. a scan or needle injection). Conversely, we excluded reviews that focused on pain management or care satisfaction outcomes only.

Lastly, our fourth criterion specified that reviews were published as full texts in peer-reviewed journals. To maximise the relevance of included systematic reviews, searches were limited to material from 2013 onwards. Indeed, these dates account for the significant advancements in immersive technology that have occurred in recent years, thereby reflecting modern XR devices that we see in the present day. However, we did not place any restrictions on the type (or date) of primary study analysed, the comparison conditions employed, or the language and region in which they were produced. We excluded conference abstracts, dissertations and theses, since these were not sufficiently detailed or accessible for meaningful evidence synthesis and/or practical application. Furthermore, we excluded articles where a target body of evidence had not been identified for a specific research question (e.g. ‘expert reviews’) or where included studies had not been synthesised.

### Databases and search strategy

Searches were undertaken on 30 May 2023 in MEDLINE, Embase and APA PsycINFO in the Ovid platform and in Epistemonikos. Our strategy incorporated terms relating to intervention, condition, study design and database-appropriate combinations of title and abstract words, subject headings and other controlled vocabularies (for full search strategies and associated number of records, see Supplementary File 1).

### Selection process

Following completion of the searches, records were deduplicated and assessed for eligibility. Here, records were screened independently and in duplicate by two separate reviewers. Firstly, each title and abstract were examined for relevance and possible inclusion, with eligible items added to a merged list of records for further inspection. Thereafter, full-text papers were obtained and screened by the review team, who each recorded any determined reasons for exclusion. Such decisions were recorded hierarchically, based on whether records contained: (i) an inappropriate study type, (ii) inadequate methods, (iii) inappropriate interventions or (iv) an inappropriate focus (i.e. for reviews that were not patient-directed or related to health and procedural anxiety). Instances of disagreement between reviewers were resolved via discussion and recourse to a third reviewer.

### Data labelling and extraction

To create a map of the research evidence, eligible reviews were labelled by a member of the review team, using a pre-defined set of descriptors. These descriptors captured the types of evidence included (e.g. effectiveness, implementation evaluations), the populations considered (e.g. children and adults), the types of outcomes addressed (e.g. general health anxiety and procedural anxiety) and the method of analysis (e.g. narrative synthesis, meta-analysis, scoping and realist synthesis) for each individual review. Included technologies were also labelled at a pooled level, with respect to the type of XR used (e.g. immersive or non-immersive devices), the method of intervention (distraction, exposure and/or education; see Box 1) and the indications for each intervention (e.g. needle-related procedures, burn and wound care). Here, immersion levels represent how fully a system engages a user's sensory and motor channels as if they were in the real-world^23^ with immersive XR devices enabling real-time, body-based interactions within a surrounding 3D environment (e.g. as in 6 Degrees of Freedom headsets) and non-immersive systems providing more limited interaction capabilities (e.g. as in 360° videos or screen-based environments). Since we were examining review-level evidence, it was unlikely that precise descriptors for each individual study intervention would be consistently reported (e.g. relating to the frequency and duration of activities). Hence, this information about XR delivery was not discretely extracted from the records. Nonetheless, common intervention formats and content types were captured as part of the main headline findings and summary details, as outlined below.

In addition to these labelling procedures, we extracted summary details from all the eligible full-text reports. Specifically, descriptions of the review objectives (e.g. the clinical context and anxiety outcomes), the number of studies evaluated and the main headline findings were documented as free text for each included study. As above, this process was performed by one reviewer, with findings then checked by a second team member and used to identify priority areas for evidence synthesis. Any disagreements between reviewers during data extraction were resolved via discussion, with recourse to a third reviewer where needed. All data extracted and/or analysed during the review are available at: https://osf.io/nhzf8/.

### Methodological quality assessment

All systematic reviews that evaluated intervention effectiveness were independently appraised by two members of the research team, using the AMSTAR-2 tool.^24^ Reviews were assessed for methodological quality across 16 evaluative domains, which include elements relating to the search strategy, justification of excluded studies, risk of bias assessments and/or consideration, and meta-analytical procedures (for full criteria, see Supplementary File 4). A rating of adequacy was provided for each domain, and a summary quality rating was provided based on the pooled data from ‘critical’ (i.e. domains 2, 4, 7, 9, 11 and 15) and ‘non-critical’ (i.e. all other domains) appraisal items. Specifically, quality was either deemed ‘critically low’ (for those containing ≥2 ‘no’ ratings on critical domains), ‘low’ (for those with ≤1 ‘no’ ratings on critical domains), ‘moderate’ (for those with ≥2 ‘no’ ratings on non-critical domains) or ‘high’ (for those with ≤1 ‘no’ on a non-critical domain) for each appraised study. The likely impact of methodological quality and bias on any results was subsequently examined in our narrative synthesis, as outlined below.

### Data synthesis

Extracted data were organised and interpreted using an evidence map based on our labelling (described above). Reviews were tabulated according to each labelled category, while headline results were extracted as free text. From here, findings were narratively synthesised within each cell of the evidence map to address the consistency, quality and range of the included evidence (e.g. in terms of pooled population characteristics, anxiety indicators, AMSTAR-2 ratings and the different intervention types/methods). As a rule, we interpreted standardised mean differences using standard thresholds for small (0.2), medium (0.5) and large (0.8) effect sizes. Finally, as an exploratory step, we calculated corrected coverage areas (CCA) for combinations of outcome, population, intervention type and indication where more than three reviews addressed a given combination. Following procedures set out by Hennessy and Johnson,^25^ we first created a citation matrix of all primary studies included for each review. Then, CCA were calculated using the following equation:

$$CCA=\frac{N-r}{(r\;\times c)-r{\;}^{\prime }}$$

where *N* is the total number of included studies across the reviews, *r* is the number of unique study publications and *c* is the number of reviews within each comparison.

## Results

### Search results

**Figure 1.**
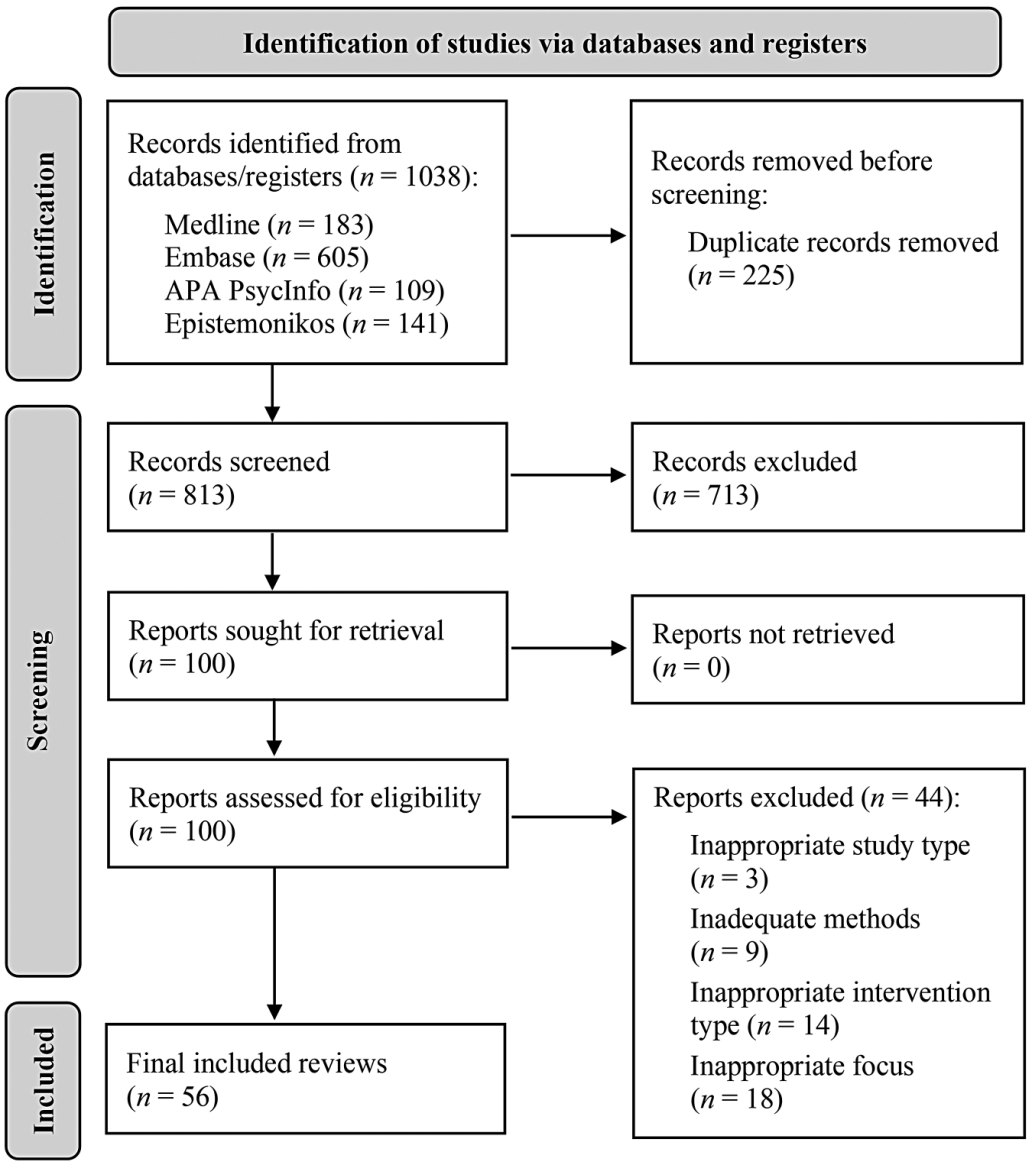
PRISMA flow chart of retrieved, screened and included articles.

Our searches yielded 1038 records, 813 of which were screened by title and abstract following de-duplication. From here, 713 records were excluded and 100 proceeded onto full-text screening. A total of 44 studies were excluded in this subsequent phase, the reasons for which are detailed in Supplementary File 2. In turn, 56 articles were deemed to meet our inclusion criteria and were subsequently analysed by the review team. A PRISMA flowchart of this study selection process is presented in Figure 1.

PRISMA: Preferred Reporting Items for Systematic Reviews and Meta-Analysis.

(Figure 1).

### Review characteristics

Included reviews were all published between the years 2018 and 2022. The majority of the reviews (*n* = 35) performed meta-analyses, though the sample contained narrative syntheses (*n* = 16) and scoping reviews (*n* = 5), which did not conduct data pooling. A summary of characteristics for each review is provided in Supplementary File 3. Notably, all of the reviews examined the effectiveness of XR interventions in some capacity; however, specific evaluation criteria and methodologies varied. Anxiety data were generally extracted from standardised measurement tools or visual analogue scales, which focused on responses from the patients (e.g. using the State-Trait Anxiety Inventory^26–28^ and Hospital Anxiety and Depression Scale^29^) and/or from suitable clinicians, caregivers and trained observers (e.g. using the Facial Affective Scale^30^ and Yale Preoperative Anxiety Scale^31,32^). Here, both trait and state anxiety outcomes were evaluated, as well as physiological indicators of anxiety (e.g. heart rate^33–36^ and skin conductance response^33^). Some reviews pooled participant data from both state and trait anxiety measures within the same analysis procedures, despite these outcomes generally being considered as distinct psychological constructs that should be examined or interpreted separately. A selection of reviews focused on wider intervention outcomes (e.g. technical performance,^37,38^ compliance^38–41^ and mechanisms of action^42^).

Most reviews (30/56) included XR as the sole reported intervention, but some incorporated XR alongside other co-interventions (e.g. music,^43^ video games,^37,44,45^ physiotherapy,^44^^,46–51^ mindfulness activities^52^ and educational material^41^^,53–56^). Analyses synthesised data from adults (in 20 reviews), children (in 19 reviews), or a combination of the two (in 17 reviews). This included participants from as young as two years old,^57^ through to elderly patients with chronic conditions.^36,43^ Population demographics were seldom reported, particularly in relation to race/ethnicity and socioeconomic data.

### Methodological quality

**Figure 2.**
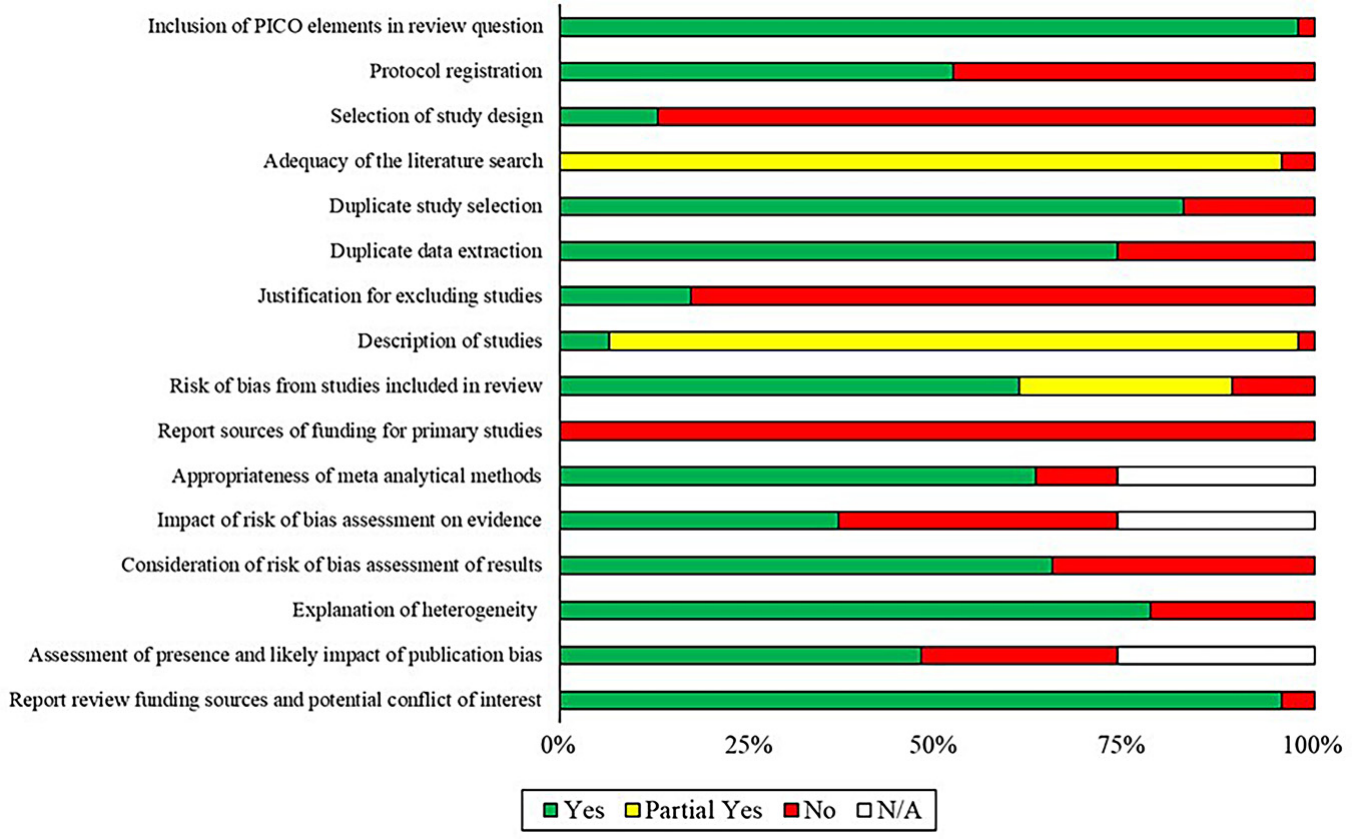
Summary of AMSTAR-2 quality appraisals. Bars illustrate the proportion of included reviews (*n* = 46) that were rated as either low risk of bias (green), showing some concerns with regard to bias (yellow), or as high risk of bias (red). White bars represent the reviews without meta-analysis, where specified criterion ratings were not applicable.

AMSTAR-2 ratings for each of the evaluated studies are summarised in Figure 2 (see Supplementary File 4 for item-level appraisal judgments of each review). Quality indicators were highly variable between the reviews, but common limitations were evident. Specifically, 22/46 of the appraised reviews did not provide evidence of their protocol being pre-registered or otherwise published, and only six^36,43^^,58–61^ were deemed to provide sufficient explanations for their selection of the study designs in their inclusion criteria. While 44/46 of the reviews searched at least two databases and reported a full search strategy, none of these performed comprehensive searches (e.g. of trial/study bibliographies, registries and grey literature) within 24 months of completing their research (and in consultation with field experts). Only three reviews^40,55,62^ were deemed to have described relevant study characteristics in detail (in terms of their populations, interventions, comparators, setting and any follow-up timeframes) and none of the articles reported on the sources of trial funding. For the summary AMSTAR-2 ratings, two reviews^34,63^ were appraised as ‘moderate’ quality, while the remaining were deemed to be of ‘low’ (*n* = 15) or ‘critically low’ (*n* = 29) quality.

(Figure 2).

### Synthesis of included systematic reviews

A detailed summary of extracted data is presented in Supplementary File 3. Reviews studied a breadth of health and procedural anxieties, from a variety of clinical contexts. An average of six studies was examined in each article, although this number spanned from 1 to 32. Overall, 45/56 reviews concluded that XR successfully reduces patient anxiety, and 9 reported mixed or inconclusive findings. Meta-analyses mostly showed large effects in support of XR (reported in 21 reviews); however, moderate (7 reviews), small (3 reviews) and null (4 reviews) findings were also documented (see Supplementary File 5), and the aforementioned limitations in methodological quality must be considered. Heterogeneity ranged from minimal (*I*^2^ = 0%^44,46,48,64^) to extremely high (*I*^2^ ≥ 95%^65–67^), indicating that interventions may not be producing a consistent clinical effect and that average pooled estimates may not be generalisable to all settings or populations. Moreover, confidence intervals were generally wide and approached zero in some cases (Supplementary File 5); hence, pooled study effects were uncertain or imprecise, even within these contexts.

Null or inconclusive pooled effects were typically attributed to limited sample sizes and methodological inconsistencies, especially in relation to anxiety measurement tools, patient characteristics and XR device selection.^39,55,66^^68–70^ A variety of different control conditions were also used across the evaluated evidence (e.g. usual care, other technology applications, and conventional education or distraction methods). Corrected covered areas for combinations of outcome, population, intervention type and indication (where more than three reviews addressed a given combination) ranged from 0.13 to 0.23. This is suggestive of very low overlap among reviews aiming to assess ostensibly the same target body of evidence and reflects that reviews were employing inconsistent criteria for classifying eligible patients, XR interventions and anxiety measurement tools. Thus, while low overlap reaffirms the breadth and diversity of research examined within this review and suggests that our findings are less likely to be affected by duplication bias, the lack of common identified evidence limits synthesis reliability and the cumulative strength of findings across evaluated studies.

#### Procedural anxiety

**Figure 3.**
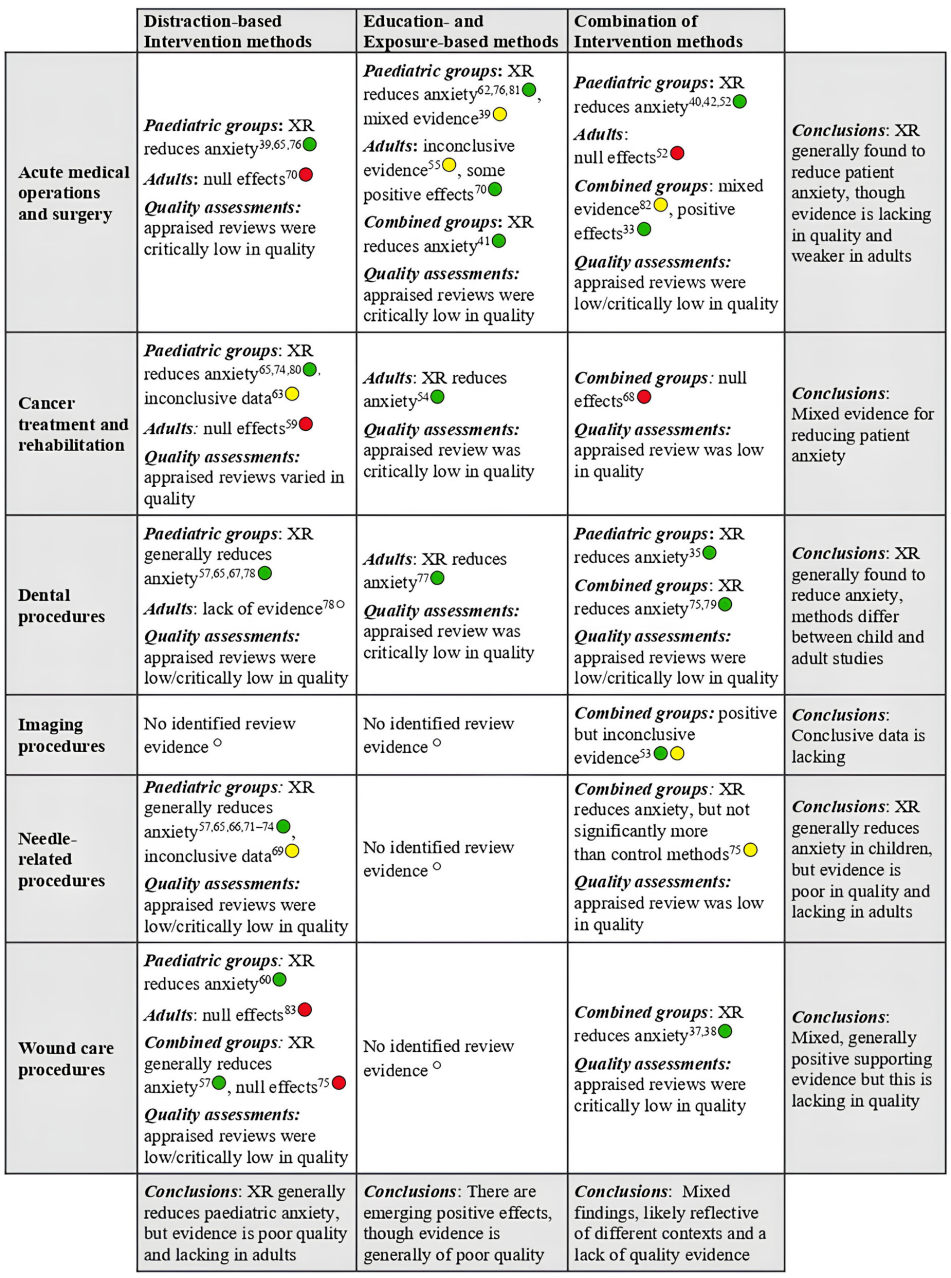
SoFT for procedural anxiety interventions. Green circles denote findings that support the efficacy of XR, although caution must still be taken before applying these technologies within clinical practice (see the Discussion section). Yellow circles highlight inconclusive evidence, and red circles signify null/undesired effects; in both cases, results do not support the implementation of XR within clinical practice. Empty circles indicate that there is a lack of research evidence within a given indication and population.

SoFT: summary of findings table; XR: extended reality.

The majority of extracted reviews (38/56) focused on specific procedural anxieties, including those relating to surgical operations, needle injection, wound care, chemotherapy, imaging and dental procedures (Figure 3). Although the mechanisms for each of these anxieties may differ, the fact that they are acute, event-specific and transitory is indicative of common underlying features, and some of the included reviews synthesised data across several of these contexts (Supplementary File 3). The most common focus of evaluation concerned the management of needle-related anxieties, which was reviewed both in isolation^66,69^^,71–73^ and within general reviews of preoperative anxiety.^42,57,65^^,74–76^

On inspection of the needle-focused reviews, positive pre- and perioperative outcomes were consistently documented for self-reported anxiety measures when compared to routine or standard care controls,^66^^,71–74^ but results were inconclusive for post-procedure anxiety scores.^69^ With regards to the other types of procedural anxiety, XR interventions were also concluded to have positive effects in relation to dental (for 6/6 reviews^34,35,67^^,77–79^), radiotherapy (1/1 review^54^), MRI scanning (1/1 review^53^) and burn wound care (3/4 reviews^37,38,60^) procedures. Reductions in chemotherapy-related anxiety were supported in syntheses of paediatric research,^74,80^ but null effects were reported in a meta-analysis of adult data,^59^ indicating that results are generally inconclusive within this clinical context (Figure 3). Mixed findings were also evident in the context of wound care procedures, with null results reported in evaluations of XR-based anxiety interventions for adults with burn injuries.^75,81^

Notably, the two reviews that received ‘moderate’ overall AMSTAR-2 ratings^34,63^ highlighted mixed findings in terms of reducing procedural anxieties; hence, evaluations that were wholly positive in support of XR were based on ‘low’ or ‘critically low’ quality evidence. Mixed findings were also documented in general reviews of procedural anxiety, which collated evidence from multiple different operations and acute care settings (see Supplementary File 3). Here, it was identified that interventions may be less effective when XR is administered by non-clinicians^41^ or when its content and equipment is not appropriate for the target user (e.g. in terms of age, gender, size/weight, or cultural factors^42,52^; see the Implementation Outcomes section). However, all five of the reviews that performed meta-analyses on miscellaneous medical procedures^40,52,62,65,76^ (see also^82^) found a significant pooled effect in favour of XR-based interventions, when compared to controls (Supplementary File 5).

(Figure 3).

#### General health anxiety

For more general health anxieties, research tended to focus on patients with long-term conditions (such as cardiovascular disease,^43,46,48^ cancer,^36,45,47^^,49–51^^,58,61,84^ and chronic pain disorders^44,64,85^) or pregnant people accessing maternity services.^86–88^ In these contexts, interventions aimed to minimise more chronic and non-specific forms of anxiety over extended periods of treatment, care and/or rehabilitation. Thus, interventions were typically longer in duration and higher in frequency than those targeting procedural anxieties (some of which involved a single session of under 5 min^75^).

**Figure 4.**
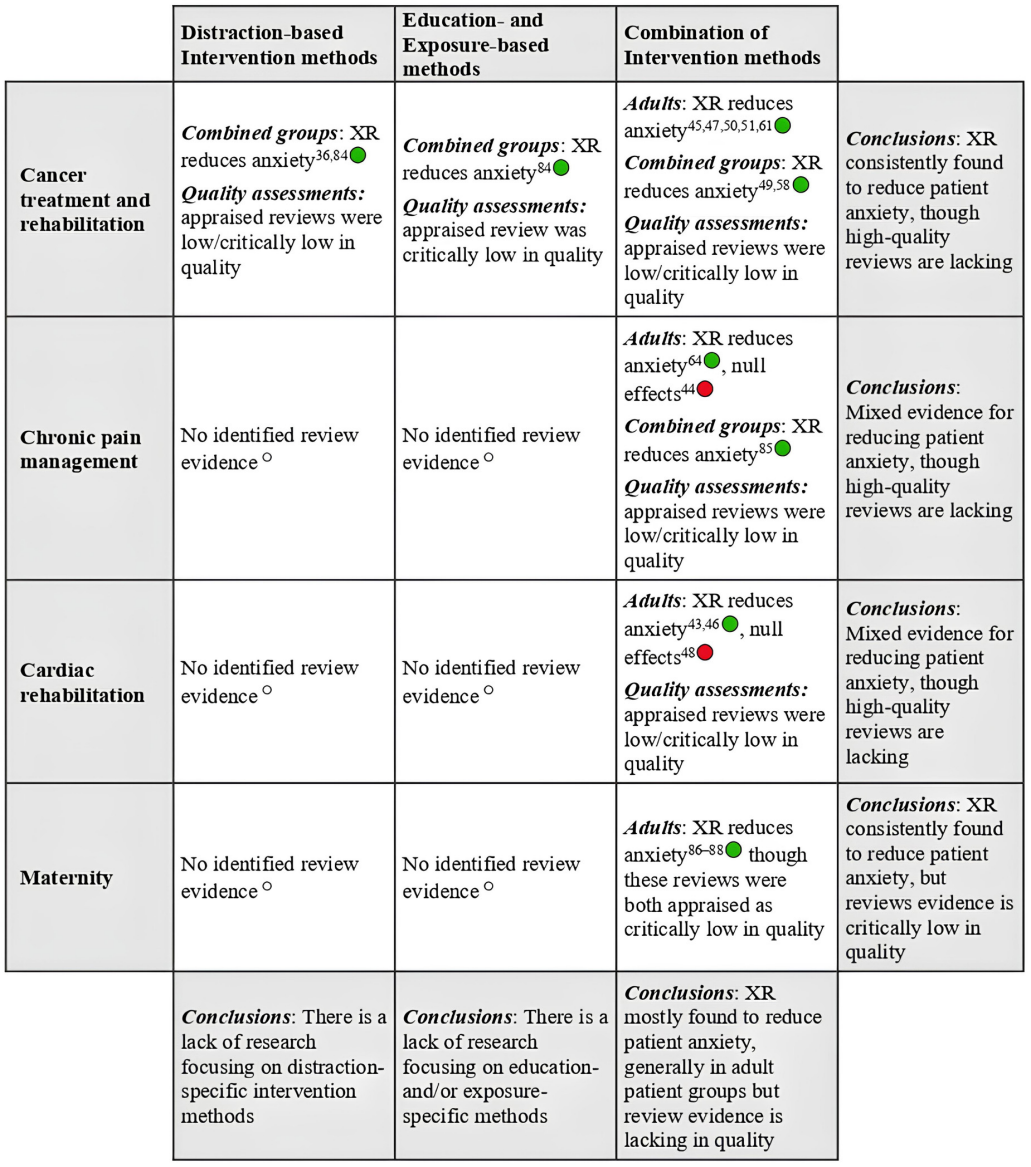
SoFT for general health anxiety interventions. Green circles denote findings that support the efficacy of XR, although caution must still be taken before applying these technologies within clinical practice (see discussion section). Red circles signify null/undesired effects, where results do not support the implementation of XR within clinical practice. Empty circles indicate that there is a lack of research evidence within a given indication and population.

SoFT: summary of findings table; XR: extended reality.

Overall, findings were relatively consistent in relation to general health anxieties, with significant reductions in anxiety supported in 16/18 reviews (Figure 4). Null findings were observed in evaluations of exercise-based therapy for adults with cardiovascular disease^48^ and chronic musculoskeletal pain.^44^ However, more recent reviews provided evidence in favour of XR-based cardiac rehabilitation,^43,46^ and significant reductions in anxiety emerged for chronic pain interventions when paediatric and adult data were pooled.^85^ While consistent in their positive evaluations, studies in cancer patients were wide-ranging in methodology, with reviews assessing a variety of state and trait anxieties within hospital treatment,^36,74^ symptom management,^45,58,61,84^ and rehabilitation^47^^49–51^ pathways. These data were frequently combined within review analyses; thus, direct comparisons between more situational (e.g. anxiety about chemotherapy at a given moment) and enduring (e.g. anxiety about recovery during long-term treatment) forms of anxiety were not sufficiently made.

Maternity-related outcomes were examined in three reviews,^86–88^ and positive changes in anxiety were appraised in each of these analyses. Again, XR interventions targeted various indications, ranging from anxieties about surgery and maternal delivery through to general mother-foetus interactions. Evidence of effectiveness was observed across these indications, but findings in one review^88^ suggested that XR is less effective in cases where anxiety occurs over longer timescales (e.g. ahead of planned caesarean section). As with all the general health reviews, it must also be noted that AMSTAR-2 quality appraisals were consistently ‘low’ or ‘critically low’ for this evidence (e.g. in terms of their overall scores: Supplementary File 4) and that widespread methodological shortcomings were evident (Figure 2).

(Figure 4).

#### Patient population

Differential effectiveness of XR interventions was first examined in relation to patient age, given the prevalent focus of this characteristic in the literature. Notably, most paediatric studies (14/19) focused on the management of surgical or needle-related procedural anxieties (Figure 3), where interventions were typically distraction-based and delivered on VR systems. While 12 of these reviews concluded that XR could successfully reduce anxiety, the remaining two^34,39^ highlighted mixed evidence from a limited study pool. Meta-regressions indicate that XR is of equal effectiveness in younger and older children.^76^ The adult-only reviews were more wide-ranging in methodology (see Figure 3 and 4), but similarly utilised VR over MR or AR systems. There was a large proportion of older age participants in some of the included reviews, especially those that focused on general health anxieties during cancer treatment and cardiac rehabilitation. That said, these reviews did not exclusively focus on older age patients and data from young adults were included. In terms of effectiveness, while 14/20 adult-focused reviews supported the use of XR, there were also null (in four reviews^44,48,59,81^) and inconclusive (in two reviews^55,70^) findings. None of the adult-focused meta-analyses directly compared anxiety outcomes between younger and older adults (e.g. using meta-regressions or subgroup analyses).

From the perspective of directly comparing intervention effectiveness data, the largest review in our analysis – a narrative synthesis of 32 studies^57^ – indicated that empirical support was weaker in adult, compared to child, populations. However, these conclusions were based on distraction-based interventions only, and positive outcomes were still observed for both groups. For many indications, there are insufficient data to compare children and adults,^78^ and methodological limitations preclude strong conclusions from being made. Nonetheless, evaluations in acute care settings suggest that XR-related reductions in procedural anxiety are less consistently observed in adults (Figure 3), potentially due to a reduced acceptability of interventions among older patients (see the Implementation Outcomes section).

Due to the aforementioned limitations in study reporting, comparisons between socio-demographic groups were not possible, and differential patterns of intervention effectiveness by population characteristics remain broadly unclear. Notably, only 2/35 of reviews^52,66^ that performed meta-analysis presented anxiety-focused subgroup comparisons or covariance analyses with descriptive participant data that was *not* age-related. Both studies included sex as a covariate in meta-regression analysis, with the proportion of female patients accounting for considerable heterogeneity in one review (a general meta-analysis of preoperative anxiety in both children and adults) but not the other (a specific meta-analysis of paediatric anxiety during venous access procedures). No subgroup comparisons or covariance analyses were performed for other sociodemographic variables, including those that are equity-relevant for health opportunities and outcomes (e.g. race/ethnicity, employment and socioeconomic status).

#### Intervention types

Wide-ranging intervention approaches and methodologies were evaluated across the reviews, both in relation to the content of XR programmes and how they were delivered. A total of 15 reviews evaluated studies of distraction-based interventions only.^34,36,57,59,60,63,66,67,69^^,71–74^^,78,80^ In these interventions, XR was typically administered in conjunction with anxiety-inducing stimuli during a single session, although various software and multimedia were utilised. Specifically, distraction methods included nature experiences (e.g. sea, forest or magical world scenes^43,57,59,63,66,67,69,72,74^), passive videos (e.g. 3D cartoons^57,63,66,67,72,74^), interactive applications (e.g. adventure games,^36,57,59,60,63,66,69,72,74^ cognitive tasks^72,80^ and roller coaster simulations^57,63,67,72,74^) and various relaxing environments (e.g. mindfulness^52^ and multisensory immersive therapy apps^43,59^). Of the 15 distraction-based analyses, 12 concluded that XR successfully reduces patient anxiety, with most interventions addressing specific procedural anxieties or phobias in children (Figure 3).

The remaining reviews typically focused on education tools (e.g. virtual operating room tour experiences and immersive informational videos), exposure therapies, rehabilitation activities, or miscellaneous XR methods. Here, XR was typically delivered before key medical procedures or it was delivered more progressively over time, as part of longer-term treatment programmes. Data relating to these interventions were typically pooled with other approaches, such as distraction-based methods, and general limitations in review methodologies were evident, as shown by low or critically low overall AMSTAR ratings (Supplementary File 4). Additionally, it was challenging to categorise interventions into different ‘types’, since a combination of methods was used within studies (e.g. immersive content that was both educational *and* exposure-based) and there were often insufficient descriptive details provided (e.g. relating to their theoretical basis or mechanisms of action). Direct quantitative comparisons of different intervention methods were not performed within the reviews. However, isolated meta-analyses of paediatric data^76^ revealed comparable moderate effects for exposure- and distraction-based XR on procedural anxiety. Moreover, reviews that were solely dedicated to exposure- or education-based methods concluded that XR lowers patient anxiety within chronic pain management,^42,85^ surgical operations,^41,76^ and radiotherapy^54^ indications (see child and adult review findings in Figures 3 and 4).

#### Technology features

Numerous different forms of XR were examined, including head-mounted display systems, smartphone viewing platforms, standalone glasses/goggles and non-immersive screen-based hardware. Despite their growing prevalence and availability across general user markets, there were no dedicated analyses for AR- or MR-based systems identified; instead, VR remained the prevalent technology in the literature. Most articles (48/56) pooled data from immersive and non-immersive platforms or did not specify immersion characteristics. This largely prevented any conclusions from being made about the influence of immersion on empirical data. When specified, non-immersive forms of XR were often used to render more passive multimedia experiences (e.g. 3D videos, relaxing nature scenes and educative tours) than the immersive XR programmes. Notably, there was preliminary evidence that immersive devices could be more effective than non-immersive devices for reducing anxiety within specific contexts (e.g. during paediatric needle or wound care procedures^57^; longer-term cancer treatment^61,63^); however, different hardware features were seldom compared in any formal sensitivity or subgroup analyses. Nonetheless, 7/56 reviews consigned their analyses to immersive XR systems only, and positive intervention outcomes (i.e. reduced patient anxiety) were reported in 4 of these cases.^61,62,76,77^

#### Implementation outcomes

A SoFT for implementation outcomes is presented in Supplementary File 6. Firstly, there were broadly high levels of acceptability and usability that emerged across clinical indications. However, evidence was typically lacking for these indications, particularly in reviews of general health anxieties and for methodological assessments (e.g. comparisons of distraction- and exposure-based approaches). Acceptance and usability issues were highlighted for specific populations; for example, it was reported that younger children can show greater difﬁculties with using XR equipment^62,79^ and that older adults may be less receptive to adopting these types of technology.^33,37^ These age-related issues could contribute to poorer intervention adherence (see^33,39,58^), with non-compliance rates reported as high as 21% in the review evidence^39^ (referring to a study of 4–5 year old children^89^). Relatedly, some patients reported discomfort or issues when wearing XR equipment, potentially due to inappropriate hardware and/or delivery components. For instance, ergonomic issues with the size and fit of headsets can present barriers for patients (particularly younger children and older/frail adults),^33,42,62,67,79^ while challenges relating to risks of falling (e.g. in elderly patients^46^) and infection control (e.g. in clinical settings^42^) are raised. From a service barriers and acceptance perspective, reviews highlighted the potential need for staff with prior training, technical skills/knowledge and time availability to administer these interventions effectively,^37,38,42,46,54,58,71,86^ though these demands may be minimal compared to alternative intervention options.^40,41,62,79^

Notably, there were few adverse effects detailed in the review articles. Indeed, while infrequent symptoms of cybersickness and/or dizziness were documented in a minority of studies included in the reviews (see^33,38,47,58,62,67,83,84,88^), the incidence of negative side effects and dropout was often at comparable rates to control interventions (or even lower in prevalence^37,40,88^). These infrequent adverse effects were not shown to be associated with any given intervention type, XR technology, or patient population, and no differential patterns of occurrence are observable across the review evidence (see Supplementary File 6).

## Discussion

Systematic reviews have catalogued the growing evidence concerning the use of XR interventions for managing health and procedural anxieties across wide-ranging clinical contexts and populations, with diverse types of intervention and technologies. In light of this array of clinical applications, findings are by no means homogenous and remain inconclusive for many specific anxiety-related indications. Notably, many published systematic reviews in this field do offer support for emerging proposals that XR interventions can successfully alleviate procedural and general health-related anxieties (see Figures 3 and 4). Nonetheless, the notable dearth of high-quality reviews available in the current literature is magnified by insufficient reporting of key study characteristics (e.g. details about the delivery of interventions and technologies used). This limits the potential exploitation of empirically supported XR programmes within current and future care pathways.

The most prevalent application of XR concerned the management of procedural anxieties (i.e. acute and excessive fears relating to specific medical procedures). Review outcomes were generally positive in this area, and various XR interventions were found to successfully reduce patient anxiety before or during treatment. Needle-related phobias emerged as a common topic of study, and meta-analyses of various clinical indicators were influenced by this prevalent research focus. Nonetheless, initially promising results emerged in relation to dental, imaging and wound care procedures (see Figure 3), and XR-based interventions were found to lower pre- and peri-operative anxieties within diverse surgical domains. While most procedural interventions sought to distract patients away from anxiety-related stimuli, numerous different exposure- or education-based methodologies were also found to elicit positive patient outcomes. Indeed, despite there being comparatively less research in these areas, emerging evidence within dental care, cancer treatment and acute surgery lends support for the approach of gradually exposing patients to anxiety-related stimuli and/or informing them about their upcoming procedure (in line with results from broader phobia and acute stress studies^14–16^). That said, supporting evidence was generally appraised to be low or critically low in quality, from a methodological perspective, and associated effect sizes were often highly heterogeneous, indicating that certain results may lack generalisable across clinical contexts or populations. Hence, our analyses illustrate promising yet inconclusive evidence for the suggestion that XR could be beneficial for reducing context-specific state anxieties, either by distracting patients during a stressful procedure or by exposing and/or educating them in advance of their medical visit.

Reviews also evaluated wide-ranging interventions for more general health anxieties, where largely positive findings again emerged, albeit from relatively poor-quality evidence. Indeed, XR was shown to reduce anxieties associated with symptom management and recurrent care procedures (e.g. chemotherapy), as well as broader anxieties experienced during extended periods of treatment. Notably, consistent positive effects were presented in reviews of maternity and cancer treatment pathways, and updated reviews of cardiac rehabilitation and chronic pain therapies provided further support in patients with long-term health conditions. However, while analyses were typically restricted to users with a shared clinical diagnosis/pathway, multiple reviews pooled outcomes relating to both state and trait anxiety. This is problematic, given that state and trait anxiety are typically considered distinct constructs,^90,91^ yet meta-analyses are designed to synthesise estimates of the same underlying true effect. Furthermore, some reviews combined data from targeted physical rehabilitation applications (e.g. ‘exergames’) with those obtained in more general distraction- and relaxation-based XR experiences. As a result, conclusions were often derived from interventions with markedly different profiles and indications. Future research should address this ‘blurring’ of conceptual features to pinpoint the specific anxiety-related indications that can be effectively managed. This is pertinent for examining general health anxieties, where there are suggestions that XR interventions may be less effective in certain contexts (e.g. in cases where anxiety builds up over long periods^88^).

Review data provided qualitatively stronger support in favour of paediatric (compared to adult) patient interventions, particularly in acute care settings and for distraction-based methods. However, conclusive differential effects by age were generally lacking in the research, particularly in relation to direct comparisons of younger versus older adults, and clear methodological shortcomings were evident (see below). Furthermore, current interpretations may be influenced by different intervention contexts and indications. Specifically, a large proportion of paediatric studies examined outcomes associated with specific medical procedures (e.g. needle-related phobias; see Figure 3), where positive changes can be successfully achieved through numerous, often simple, intervention methods (e.g. using engaging videos/games or generic nature experiences). Empirical support did emerge for adult-centred XR interventions that addressed comparable anxiety indicators (e.g. for patients with phobias about acute dental or imaging procedures). However, research is required into possible age-related differences that could impact XR's long-term efficacy.

When examining age- and technology-related factors, consideration should be placed on broader implementation outcomes, such as acceptability and usability, which are infrequently evaluated in the literature. Reviews suggested that the acceptability of XR may be lower among older patients, and that generic anxieties about technology could lead to negative intervention outcomes.^33^ Similarly, challenges with usability and discomfort have been documented when implementing XR with young children.^62,79^ It must be noted that most current evaluations indicate generally high levels of acceptance and usability within paediatric and adult populations (see Supplementary File 6), and that these poorer outcomes for young children and elderly adults correspond with patterns seen in wider areas (e.g. in the immersive media industry^92^ and in more general health-related sectors^93^). Moreover, the low prevalence of adverse effects supports the safety and feasibility of XR implementation across broad populations. However, our results emphasise the importance of developing age- and content-appropriate programmes for diverse user groups. Future innovations should focus on how XR interventions can be effectively tailored to these different service groups, by potentially modifying hardware (e.g. the size and functionalities of headset devices) and exploring the unique capacity to personalise immersive content (e.g. see adaptive XR and affective computing concepts^94–96^). Longitudinal research studies are also recommended, which focus on the durability of intervention outcomes (e.g. at multiple follow-up timepoints), as well as their sustainability in practice (from a patient and service perspective).

VR technologies were far more prevalent than AR and MR in the research, although various devices were applied (e.g. head-mounted displays, standalone glasses and mobile- or PC-based systems). Given the growing advancements in AR and MR systems, research is undoubtedly required to identify the optimal methods of delivering simulation therapies. As technology innovations are increasingly blurring the lines between the three different types of XR, it is important that precise hardware features and multisensory stimuli within an intervention are specified (and not simply describing it as a ‘VR game’ or ‘immersive virtual experience’). Relatedly, limitations in study reporting mean that there is little clarity on any focal intervention components. Interventions could be broadly classified into distraction methods, which have commonly proven effective in reducing procedural anxieties, and a variety of education-, exposure- or rehabilitation-based methods, which have been evaluated less extensively. Multiple forms of intervention were often combined within reviews (and sometimes within trials), making methodological comparisons uncertain and challenging to perform. Furthermore, interventions could often be attributed to diverse mechanisms of action. For instance, data from distraction-based methods seeking to provide fun and engagement were often pooled with those designed to elicit calm and relaxing states, while preoperative interventions often combined educational information with exposure-like simulation experiences. There is also variety in the intended effects of the intervention, with some designed to ensure the patient is compliant to a programme (e.g. by attending appointments, remaining still and/or enduring prolonged medical procedures), and others focusing more specifically on their emotional experiences (e.g. ensuring they are happy and not distressed). While it is possible that some of these varying methods share common or complementary mechanisms of action (e.g. in terms of attentional diversion, fear activation and neural habituation; see^20,97^), current understanding is limited. Hence, it remains unclear precisely which benefit pathways should be targeted in future applications.

A related technological issue was that, while identified as a predictor of positive study outcomes, most reviews did not discriminate between immersive and non-immersive XR in their analyses. Those who restricted their studies to immersive devices only tended to employ inconsistent definitions and/or inclusion criteria for this variable. While reflective of a broader issue in XR research, these inconsistencies add additional uncertainty to the wider assessment of anxiety interventions. Future research should thus explore how immersion affects patient outcomes across different contexts and indications. A specific analysis of *distraction-based* interventions highlighted that immersive XR could be more effective than non-immersive devices for reducing patient anxiety.^63^ These results support the notion that immersive technologies hold greater ‘distraction power’^50^ in health-based interventions, potentially through fostering a greater sense of presence (i.e. feelings of existing in a virtual environment). By examining what levels of immersion are required for different patient groups, one can shed light on the fundamental mechanisms of action that underpin successful interventions and the technical conditions that are needed to capture these processes.

Most reviews were appraised as low or critically low in quality, and widespread limitations emerged in relation to search procedures, registration and the reporting of methodological information. Notably, only 3/46 of the appraised reviews^40,55,62^ comprehensively described studies with respect to their interventions, clinical setting, population(s) and comparator conditions (as per AMSTAR-2 criteria^24^). This lack of detail impairs our ability to evaluate key features, such as focal delivery components and contexts associated with positive outcomes. For example, it is unviable to draw conclusions about the optimal duration, frequency, or timing of XR based on our extracted data. However, such information is not only lacking in the review evidence; a large proportion of primary studies are also low in quality and fail to report essential methodological details. Moreover, anxiety measurements are often poorly defined or inappropriate for the analytical approach adopted. Both trials and reviews exhibit a dependency on simplistic visual analogue scales (which are not appended or cited) and a mischaracterisation or conceptual ‘blurring’ of state and trait anxieties (which concern fundamentally distinct neuropsychological processes).

As such, the evaluated research demonstrates significant imprecision overall; not just in the reporting of intervention methods and their intended benefit pathways, but also in relation to the broader conceptualisation and measurement of anxiety outcomes. Though unclear, these limitations may relate to the relative novelty of XR interventions in the field, which has often lent itself to a lack of established technology-specific guidelines (e.g. for study designs or reporting) and a general abundance of early-stage empirical assessments (e.g. pilot and validation studies, as opposed to randomised controlled trials or large-scale implementation evaluations).^98,99^ As the technological landscape continues to develop in the coming years, it is vital that research methods are also upgraded to meet necessary clinical and empirical standards. Indeed, as a priority for researchers in this field, we recommend that prospective studies adhere to standard protocols for assessment (e.g. using well-established and validated measurement tools) and reporting data (e.g. in line with PRISMA and AMSTAR-2 checklists), while addressing prominent methodological rigour concerns to improve confidence in results (e.g. by pre-registering protocols, studying funding sources, and enacting eligibility criteria for specific anxiety outcomes or active control groups). We also recommend that research addresses the lack of evidence surrounding modern AR and MR devices, given their emerging prevalence and availability across public sectors.

In addition to these methodological concerns, reporting is particularly limited for equity-relevant population characteristics, such as employment, race/ethnicity, community context and socioeconomic status. This is significant, given the risks and opportunities that XR poses for expanding access to healthcare. Indeed, while XR-based anxiety interventions could uniquely benefit patients who are currently under-served within healthcare systems (e.g. people with remote access to services or with specific support needs), there is also potential for inequalities to worsen if technology is improperly adopted (see recent equity synthesis^19^). There is a scarce analysis of these issues within the review evidence, meaning that current understanding of socioeconomic barriers and moderators is lacking and any differential effectiveness of XR interventions that may exist between individuals and populations remains unclear. Thus, it is imperative that future work supplies detailed and replicable information about intervention methods and equity-relevant population characteristics (see TIDIeR reporting guidelines^100^). Until possible access barriers and disparities are addressed more comprehensively, the implications for clinical practice are clear: that XR interventions should be implemented cautiously across services for the management of health and procedural anxieties. More specifically, we recommend that the growing opportunities and positive study findings presented for XR technologies are considered pragmatically in relation to equity-relevant issues and methodological limitations that are simultaneously prevalent across the evidence.

The strength of this review lies in its broad scope and transparent methodology, which offer important implications for future work. Our pre-registered analyses synthesised data from wide-ranging contexts and interventions, thus providing a holistic assessment of XR technologies for the management of health and procedural anxiety. Research and practitioners can draw upon our findings to design prospective service programmes. Indeed, despite the widespread study limitations, there is a notable volume of support emerging for XR interventions within the field, which appears to transcend multiple different indications and populations. That is not to say that XR will improve service outcomes in all contexts and patient groups; rather, our analyses have highlighted that successful reductions in health and procedural anxiety can be attained for diverse methods (e.g. distraction- or exposure-based applications), technologies (e.g. immersive and non-immersive XR), populations (e.g. children and adults) and clinical settings (e.g. during acute medical procedures or longer-term treatment pathways). Our umbrella review distinctively outlines this emerging research consensus, from fragmented and heterogeneous specialisms where conclusive findings are otherwise unclear. Crucially, it also highlights clear gaps and limitations in the research, which should inform future trials and interventions (as outlined above).

From a limitation perspective, our analyses were constrained by the lack of high-quality evidence available in the literature. Indeed, considerable weaknesses in both the review- and trial-level research restricted our ability to synthesise findings and evaluate different intervention approaches. Secondly, while our analyses of corrected covered areas implied low prevalence of overlaps (for certain combinations of outcome, population and intervention), several primary trials will have been included in multiple reviews and could have asserted greater weightings on our conclusions. These undue weightings can be avoided in future analyses through deduplication at an individual trial level and pooling of quantitative data across multiple reviews. Finally, it is possible that some potentially relevant research was not captured in our ‘umbrella review’ methodology. Although systematic searches were conducted across four comprehensive and multidisciplinary databases, additional reviews may have been identified from more clinically specific and/or regionally focused directories. However, the scope of this review was not to provide an exhaustive summary or re-analysis of study data. Rather, our methods were designed to supply a holistic overview of XR intervention studies from across diverse clinical contexts.

## Conclusion

In conclusion, the present research synthesised evidence from 56 systematic reviews, which examined diverse XR interventions for the management of health and procedural anxiety. Results highlighted the increasing volume of evidence across wide-ranging clinical contexts, particularly in relation to distraction-based interventions, and studies of paediatric patient groups and/or acute procedural phobias (e.g. needle-related anxieties or preparation for surgical operations). The majority of published reviews support the use of XR for reducing patient anxieties; however, evidence is currently lacking in quality and must be generalised with caution. Indeed, while positive outcomes have been presented in relation to both procedure-specific and more general health anxieties, and in relation to various different methodologies (e.g. distraction-, exposure- and education-based applications), there are insufficient data to identify the optimal methods, technologies and populations for future practice. Prospective research must therefore examine the effectiveness of XR interventions within integrated care and longer-term programmes, focusing on technical- and implementation-related components that impact service outcomes across pervasive health contexts and indications. Until then, we recommend that clinical practice follows a cautious approach to application, whereby emerging findings about the general utility of XR technologies are considered pragmatically alongside current methodological shortcomings and equity-relevant issues in the field.

## Supplemental Material

sj-pdf-1-dhj-10.1177_20552076251411512 - Supplemental material for Extended reality interventions for health and procedural anxiety: An overview of reviews Supplemental material, sj-pdf-1-dhj-10.1177_20552076251411512 for Extended reality interventions for health and procedural anxiety: An overview of reviews by Tom Arthur, Sophie Robinson, David Harris, Mark Wilson, Samuel Vine and GJ Melendez-Torres in DIGITAL HEALTH

sj-pdf-2-dhj-10.1177_20552076251411512 - Supplemental material for Extended reality interventions for health and procedural anxiety: An overview of reviews Supplemental material, sj-pdf-2-dhj-10.1177_20552076251411512 for Extended reality interventions for health and procedural anxiety: An overview of reviews by Tom Arthur, Sophie Robinson, David Harris, Mark Wilson, Samuel Vine and GJ Melendez-Torres in DIGITAL HEALTH

sj-pdf-3-dhj-10.1177_20552076251411512 - Supplemental material for Extended reality interventions for health and procedural anxiety: An overview of reviews Supplemental material, sj-pdf-3-dhj-10.1177_20552076251411512 for Extended reality interventions for health and procedural anxiety: An overview of reviews by Tom Arthur, Sophie Robinson, David Harris, Mark Wilson, Samuel Vine and GJ Melendez-Torres in DIGITAL HEALTH

sj-pdf-4-dhj-10.1177_20552076251411512 - Supplemental material for Extended reality interventions for health and procedural anxiety: An overview of reviews Supplemental material, sj-pdf-4-dhj-10.1177_20552076251411512 for Extended reality interventions for health and procedural anxiety: An overview of reviews by Tom Arthur, Sophie Robinson, David Harris, Mark Wilson, Samuel Vine and GJ Melendez-Torres in DIGITAL HEALTH

sj-pdf-5-dhj-10.1177_20552076251411512 - Supplemental material for Extended reality interventions for health and procedural anxiety: An overview of reviews Supplemental material, sj-pdf-5-dhj-10.1177_20552076251411512 for Extended reality interventions for health and procedural anxiety: An overview of reviews by Tom Arthur, Sophie Robinson, David Harris, Mark Wilson, Samuel Vine and GJ Melendez-Torres in DIGITAL HEALTH

sj-pdf-6-dhj-10.1177_20552076251411512 - Supplemental material for Extended reality interventions for health and procedural anxiety: An overview of reviews Supplemental material, sj-pdf-6-dhj-10.1177_20552076251411512 for Extended reality interventions for health and procedural anxiety: An overview of reviews by Tom Arthur, Sophie Robinson, David Harris, Mark Wilson, Samuel Vine and GJ Melendez-Torres in DIGITAL HEALTH

sj-pdf-7-dhj-10.1177_20552076251411512 - Supplemental material for Extended reality interventions for health and procedural anxiety: An overview of reviews Supplemental material, sj-pdf-7-dhj-10.1177_20552076251411512 for Extended reality interventions for health and procedural anxiety: An overview of reviews by Tom Arthur, Sophie Robinson, David Harris, Mark Wilson, Samuel Vine and GJ Melendez-Torres in DIGITAL HEALTH

sj-pdf-8-dhj-10.1177_20552076251411512 - Supplemental material for Extended reality interventions for health and procedural anxiety: An overview of reviews Supplemental material, sj-pdf-8-dhj-10.1177_20552076251411512 for Extended reality interventions for health and procedural anxiety: An overview of reviews by Tom Arthur, Sophie Robinson, David Harris, Mark Wilson, Samuel Vine and GJ Melendez-Torres in DIGITAL HEALTH
