# Supplementary material for: Extended reality interventions for health and procedural anxiety: An overview of reviews
Source: Digit Health. 2026 Feb 11;12:20552076251411512. doi: 10.1177/20552076251411512 (PMC12901853; doi:10.1177/20552076251411512)
Supplement: sj-pdf-1-dhj-10.1177_20552076251411512 - Supplemental material for Extended reality interventions for health and procedural anxiety: An overview of reviews [file sj-pdf-1-dhj-10.1177_20552076251411512.pdf]

Supplementary File 1. Search methods.

All searches were carried out on 30 May 2023.

MEDLINE ALL (1946 to May 25, 2023)

1	exp virtual reality/	5574
2	Virtual Reality Exposure Therapy/	877
3	Augmented Reality/	1137
4	(VR or 'virtual realit*').tw.	21365
5	('extend* realit*' or XR).tw.	3209
6	(haptic adj2 technolog*).tw.	140
7	(VRCBT or VR-CBT or "virtual exposure" or "immersive technolog*").tw.	197
8	("automated therap*" or "VR therap*" or "VR cognitive therap*" or "virtual reality therap*" or "virtual reality exposure" or VRET or "virtual reality based exposure" or VRBET).tw.	625
9	("extended realit*" or "augmented realit*" or "mixed realit*").tw.	4570
10	or/1-9	29424
11	((phobi* or anxi* or fear*) adj3 (health* or 'procedure*' or 'treatment*' or 'medical')).tw.	22709
12	exp Anxiety/	110728
13	exp Anxiety Disorders/	90047
14	exp Phobic Disorders/	14134
15	11 or 12 or 13 or 14	197719
16	10 and 15	1080
17	review*.ab,ti. or review.pt. or "systematic review"/ or overview*.ab,ti. or meta-analy*.ab,ti. or metaanaly*.ab,ti. or metanaly*.ab,ti. or meta-analysis.pt. or Meta-Analysis/ or meta-regression*.ab,ti. or metaregression*.ab,ti. or (meta adj regression*).ab,ti. or search*.ab. or synthes*.ab,ti. or metasynthes*.ab,ti. or meta-synthes*.ab,ti. or metaethnograph*.ab,ti. or meta-ethnograph*.ab,ti.	5795470
18	(letter or comment or editorial).pt.	2161472
19	17 not 18	5719251
20	16 and 19	261
21	limit 20 to yr="2013 -Current"	183

Embase (1974 to 2023 May 25)

1	exp virtual reality/	25957
2	Virtual Reality Exposure Therapy/	929
3	Augmented Reality/	2247
4	(VR or 'virtual realit*').tw.	29372
5	('extend* realit*' or XR).tw.	6937
6	(haptic adj2 technolog*).tw.	167
7	(VRCBT or VR-CBT or "virtual exposure" or "immersive technolog*").tw.	229
8	("automated therap*" or "VR therap*" or "VR cognitive therap*" or "virtual reality therap*" or "virtual reality exposure" or VRET or "virtual reality based exposure" or VRBET).tw.	830
9	("extended realit*" or "augmented realit*" or "mixed realit*").tw.	5464
10	or/1-9	51901

11	((phobi* or anxi* or fear*) adj3 (health* or 'procedure*' or 'treatment*' or 'medical')).tw.	31606
12	exp Anxiety/	287499
13	exp Anxiety Disorder/	315140
14	exp Phobia/	36664
15	11 or 12 or 13 or 14	559908
16	10 and 15	3402
17	review*.ab,ti. or review.pt. or "systematic review"/ or overview*.ab,ti. or meta-analy*.ab,ti. or metaanaly*.ab,ti. or metanaly*.ab,ti. or meta-analysis.pt. or Meta-Analysis/ or meta-regression*.ab,ti. or metaregression*.ab,ti. or (meta adj regression*).ab,ti. or search*.ab. or synthes*.ab,ti. or metasynthes*.ab,ti. or meta-synthes*.ab,ti. or metaethnograph*.ab,ti. or meta-ethnograph*.ab,ti.	6981657
18	(letter or editorial).pt. or ((animal/ or nonhuman/) not exp human/)	8480445
19	17 not 18	6183558
20	16 and 19	911
21	limit 20 to yr="2013 -Current"	605

APA PsycInfo (1806 to May Week 4 2023)

1	exp virtual reality/	11576
2	Virtual Reality Exposure Therapy/	257
3	Augmented Reality/	947
4	(VR or 'virtual realit*').tw.	10464
5	('extend* realit*' or XR).tw.	792
6	(haptic adj2 technolog*).tw.	49
7	(VRCBT or VR-CBT or "virtual exposure" or "immersive technolog*").tw.	197
8	("automated therap*" or "VR therap*" or "VR cognitive therap*" or "virtual reality therap*" or "virtual reality exposure" or VRET or "virtual reality based exposure" or VRBET).tw.	666
9	("extended realit*" or "augmented realit*" or "mixed realit*").tw.	1663
10	or/1-9	16662
11	((phobi* or anxi* or fear*) adj3 (health* or 'procedure*' or 'treatment*' or 'medical')).tw.	19696
12	exp Anxiety/	89017
13	exp Anxiety Disorders/	42535
14	exp Phobias/ or Health Anxiety/	14939
15	11 or 12 or 13 or 14	130603
16	10 and 15	990
17	exp "Systematic Review"/ or (review* or overview* or meta-analy* or metaanaly* or metanaly*).ab,ti. or "Meta Analysis"/ or meta-regression*.ab,ti. or metaregression*.ab,ti. or (meta adj regression*).ab,ti. or search*.ab. or synthes*.ab,ti. or metasynthes*.ab,ti. or meta-synthes*.ab,ti. or metaethnograph*.ab,ti. or meta-ethnograph*.ab,ti. or ("0800" or "0830" or "1200" or "1300").md.	791312
18	(comment reply or editorial or letter or "review book" or "review media" or "review software other").dt. or (electronic collection or dissertation abstract or encyclopedia).pt.	882960
19	17 not 18	591316
20	16 and 19	182
21	limit 20 to yr="2013 -Current"	109

Epistemonikos

((title:(VR OR "virtual realit*" OR "virtual exposure*" OR "extended realit*" OR XR OR "haptic technolog*" OR VRCBT OR "immersive technolog*" OR "automated therap*" OR VRBET OR VRET OR "augmented realit*" OR "mixed realit*")) OR (abstract:(VR OR "virtual realit*" OR "virtual exposure*" OR "extended realit*" OR XR OR "haptic technolog*" OR VRCBT OR "immersive technolog*" OR "automated therap*" OR VRBET OR VRET OR "augmented realit*" OR "mixed realit*")) AND (title:(anxiet* OR phobi* OR fear*) OR abstract:(anxiet* OR phobi* OR fear*)))

Limit to Systematic Reviews	141
