# Supplementary material for: Extended reality interventions for health and procedural anxiety: An overview of reviews
Source: Digit Health. 2026 Feb 11;12:20552076251411512. doi: 10.1177/20552076251411512 (PMC12901853; doi:10.1177/20552076251411512)
Supplement: sj-pdf-2-dhj-10.1177_20552076251411512 - Supplemental material for Extended reality interventions for health and procedural anxiety: An overview of reviews [file sj-pdf-2-dhj-10.1177_20552076251411512.pdf]

Supplementary File 2. List of excluded full texts with reasons.

**Authors (Year)**	**Article Title**	**Exclusion Reason**
Aguero-Millan *et al.* (2023)	Efficacy of nonpharmacologic interventions in preoperative anxiety: A systematic review of systematic reviews	Inappropriate intervention (no discrete XR user data)
Akkan *et al.* (2022)	The effect of virtual reality-based therapy on fear of falling in multiple sclerosis: a systematic review and meta-analysis	Inappropriate focus (not for health or procedural anxiety)
Alanazi *et al.* (2022)	The impact of Virtual Reality in enhancing the quality of life of pediatric oncology patients	Inappropriate methods (not a systematic review - no inclusion criteria)
Amiri *et al.* (2019)	Application of distraction techniques in obstetrics and gynaecology: a systematic review	Inappropriate intervention (no discrete XR user data)
Botella *et al.* (2015)	Virtual Reality exposure-based therapy for the treatment of post-traumatic stress disorder: a review of its efficacy, the adequacy of the treatment protocol, and its acceptability	Inappropriate focus (not for health or procedural anxiety)
Boyce *et al.* (2022)	Can Virtual Reality enhance the patient experience during awake invasive procedures? A systematic review and meta-analysis of randomised controlled trials	Inappropriate study type (not a full-text within specified date range)
Burkhart *et al.* (2023)	Interventions to reduce pediatric anxiety during orthopaedic cast room procedures: a systematic and critical analysis review	Inappropriate intervention (no discrete XR user data)
Chirico *et al.* (2016)	Virtual Reality in health system: beyond entertainment. a mini-review on the efficacy of VR during cancer treatment	Inappropriate focus (not for health or procedural anxiety)
Czech *et al.* (2022)	Virtual Reality intervention as a support method during wound care and rehabilitation after burns: a systematic review and meta-analysis	Inappropriate focus (not for health or procedural anxiety)
DeStefano *et al.* (2019)	Fear and anxiety managing methods during dental treatments: a systematic review of recent data	Inappropriate intervention (no discrete XR user data)
Dehghan *et al.* (2022)	The effect of Virtual Reality on emotional response and symptoms provocation in patients with OCD: a systematic review and meta-analysis	Inappropriate focus (not for health or procedural anxiety)
Di Guardo and Palumbo (2022)	Immersive Virtual Reality as tool to reduce anxiety during embryo transfer	Inappropriate methods (not a systematic review – insufficient synthesis)
Donnelly *et al.* (2021)	Virtual Reality for the treatment of anxiety disorders: a scoping review	Inappropriate focus (not for health or procedural anxiety)

Falguiere *et al.* (2021)	Contribution of Virtual Reality in oral surgery: a literature review	Inappropriate methods (not a systematic review – insufficient synthesis)
Gega *et al.* (2022)	Digital interventions in mental health: evidence syntheses and economic modelling	Inappropriate intervention (no discrete XR user data)
Gendia *et al.* (2022)	The use of Virtual Reality to improve patients' experience in colonoscopy: a literature review	Inappropriate study type (not a full-text within specified date range)
Gendia *et al.* (2022)	Can Virtual Reality technology be considered as a part of the surgical care pathway?	Inappropriate methods (not a systematic review – insufficient synthesis)
Gujjar *et al.* (2019)	Are technology-based interventions effective in reducing dental anxiety in children and adults? a systematic review	Inappropriate intervention (no discrete XR user data)
Harper *et al.* (2022)	The use of virtual reality in non-burn dermatological care - a review of the literature	Inappropriate methods (not a systematic review – insufficient synthesis)
Hitching *et al.* (2023)	The emerging role of Virtual Reality as an adjunct to procedural sedation and anesthesia: a narrative review	Inappropriate methods (not a systematic review - inadequate searches)
Hornsby *et al.* (2020)	Psychosocial interventions targeting recovery in child and adolescent burns: a systematic review	Inappropriate intervention (no discrete XR user data)
Hudson *et al.* (2022)	Scoping review: How is Virtual Reality being used as a tool to support the experience of undergoing Magnetic Resonance Imaging?	Inappropriate methods (not a systematic review - no specified inclusion criteria)
Indovina *et al.* (2018)	Virtual Reality as a distraction intervention to relieve pain and distress during medical procedures	Inappropriate methods (not a systematic review - inadequate searches)
Ioannou *et al.* (2020)	Virtual Reality and symptoms management of anxiety, depression, fatigue, and pain: a systematic review	Inappropriate focus (for not health or procedural anxiety)
Joda *et al.* (2019)	Augmented and Virtual Reality in dental medicine: A systematic review	Inappropriate focus (not patient-directed XR)
Lim *et al.* (2023)	Integrative review of non-pharmacological intervention and multidimensional evaluation for intraoperative anxiety under spinal anaesthesia	Inappropriate intervention (no discrete XR user data)
Lopez-Rodriguez *et al.* (2020)	New technologies to improve pain, anxiety and depression in children and adolescents with cancer: a systematic review	Inappropriate intervention (no discrete XR user data)
Maples-Keller *et al.* (2017)	The use of Virtual Reality technology in the treatment of anxiety and other psychiatric disorders	Inappropriate methods (not a systematic review – insufficient synthesis)

McCahill *et al.* (2021)	Use of Virtual Reality for minor procedures in the Emergency Department: a scoping review	Inappropriate intervention (no discrete XR user data)
Moriconi *et al.* (2022)	Effectiveness of Virtual Reality (VR) in reducing distress in children and adolescents with cancer: a systematic review.	Inappropriate focus (not for health or procedural anxiety)
Morris *et al.* (2009)	The effectiveness of Virtual Reality on reducing pain and anxiety in burn injury patients: a systematic review	Inappropriate study type (not a full-text within specified date range)
Nolet *et al.* (2020)	The adoption of new treatment modalities by health professionals and the relative weight of empirical evidence in favor of virtual reality exposure vs mindfulness in the treatment of anxiety disorders	Inappropriate focus (not for health or procedural anxiety)
Parmigiani *et al.* (2022)	Virtual reality interventions for victims of crime: A systematic review	Inappropriate focus (not for health or procedural anxiety)
Pourmand *et al.* (2017)	Emerging utility of Virtual Reality as a multidisciplinary tool in clinical medicine	Inappropriate focus (not for health or procedural anxiety)
Ridout *et al.* (2021)	Effectiveness of Virtual Reality interventions for adolescent patients in hospital settings: systematic review	Inappropriate focus (not for health or procedural anxiety)
Sajeev *et al.* (2021)	Interactive video games to reduce paediatric procedural pain and anxiety: a systematic review and meta-analysis	Inappropriate intervention (no discrete XR user data)
Scheffler *et al.* (2018)	Efficacy of non-pharmacological interventions for procedural pain relief in adults undergoing burn wound care: a systematic review and meta-analysis of randomized controlled trials	Inappropriate focus (not for health or procedural anxiety)
Suleiman-Martos *et al.* (2022)	Effect of a game-based intervention on preoperative pain and anxiety in children: a systematic review and meta-analysis	Inappropriate intervention (no discrete XR user data)
Thangavelu *et al.* (2022)	Designing Virtual Reality assisted psychotherapy for anxiety in older adults living with Parkinson's disease: integrating literature for scoping	Inappropriate intervention (no discrete XR user data)
Urlings *et al.* (2022)	The role and effectiveness of augmented reality in patient education: a systematic review of the literature	Inappropriate focus (not for health or procedural anxiety)
Van der Kruk *et al.* (2022)	Virtual Reality as a patient education tool in healthcare: a scoping review	Inappropriate focus (not for health or procedural anxiety)
Wong *et al.* (2022)	Effectiveness of Virtual Reality-based interventions for managing chronic pain on pain reduction, anxiety, depression and mood: a systematic review	Inappropriate intervention (no discrete XR user data)

Yazdipour *et al.* (2023)	Opportunities and challenges of Virtual Reality-based interventions for patients with breast cancer: a systematic review	Inappropriate focus (not for health or procedural anxiety)
Zasadzka *et al.* (2021)	Virtual Reality as a promising tool supporting oncological treatment in breast cancer	Inappropriate focus (not for health or procedural anxiety)
