# Supplementary material for: Extended reality interventions for health and procedural anxiety: An overview of reviews
Source: Digit Health. 2026 Feb 11;12:20552076251411512. doi: 10.1177/20552076251411512 (PMC12901853; doi:10.1177/20552076251411512)
Supplement: sj-pdf-3-dhj-10.1177_20552076251411512 - Supplemental material for Extended reality interventions for health and procedural anxiety: An overview of reviews [file sj-pdf-3-dhj-10.1177_20552076251411512.pdf]

Supplementary File 3. Summary of Extracted Review Data.

Reviews of Procedural Anxiety Studies

**Indication**	**Review Article**	**Review Type**	**Included Populations**	**No. of studies**	**Types of Interventions**	**Extracted Findings**
Acute medical operations and surgery	Alqudimat *et al.* (2021)	Narrative Synthesis	Children undergoing various surgical procedures	8	All relevant intervention types were included	VR was generally effective and safe for reducing peri-operative anxiety. Significant reductions were found in the majority of studies, most of which focused on exposure therapy. Limitations were identified relating to the size/weight of devices, infection control, and the availability of age- or culturally-appropriate tools.
Acute medical operations and surgery	Chen *et al.* (2023)	Meta-analysis	Children having surgery under general anaesthetic	5	Education-/exposure-based methods (all immersive forms of VR)	Meta-analysis revealed a large difference between VR and control interventions for alleviating preoperative anxiety. High levels of heterogeneity were observed and results indicated potential for publication bias (which was linked to small sample sizes). Adverse effects were not reported in the majority of included studies.
Acute medical operations and surgery	Desmet *et al.* (2021)	Narrative Synthesis	Children having surgery under general anaesthetic	4	All relevant intervention types were included	Review highlighted mixed results for VR interventions reducing preoperative anxiety in paediatric patients. Null effects were observed for some evaluated interventions, and non-compliance rates were high for exposure- and distraction-based methods.
Acute medical operations and surgery	Gaina *et al.* (2022)	Narrative Synthesis	Adults undergoing colonoscopy procedures	4	Immersive forms of VR	Synthesis highlighted limited evidence for VR reducing procedural anxiety, but potential for future implementation. Distraction-based VR did not significantly lower anxiety, though education-based applications showed positive effects. Patients had a positive overall perception regarding the VR intervention, however studies were limited in quality and often used low-end, non-interactive devices.
Acute medical operations and surgery	Gomez-Busto & Ortiz (2020)	Narrative Synthesis	Children undergoing surgery	1	Education-/exposure-based methods	Review evaluated one VR intervention study that demonstrated reductions in self-reported anxiety about abdominal surgery. Changes were significantly greater than those in control conditions.
Acute medical operations and surgery	Koo *et al.* (2020)	Meta-analysis	Adults and children undergoing surgery	10	All relevant intervention types were included	Preoperative anxiety was significantly lower in VR than in control groups. These moderate effects proved stable and had a low risk of publication bias. Meta-regressions identified that the proportion of female patients accounted for heterogenous effects, while there was a trend toward a null effects as data cumulated by year.

Acute medical operations and surgery	Simonetti *et al.* (2022)	Meta-analysis	Children undergoing elective surgery	6	All relevant intervention types were included	A small intervention effect was observed, which supported the use of VR in reducing anxiety. Heterogeneity and risk of bias was low, though there were notable methodological limitations for the studies. Compliance to VR interventions was either greater or equal to that of control conditions, though rates varied between studies.
Acute medical operations and surgery	Smith *et al.* (2020)	Narrative Synthesis	Children and adults with acute medical condition or procedures	8	All relevant intervention types were included	Found mixed evidence of utilising VR for the reduction of anxiety. Half of the included studies observed a significant reduction in anxiety, though none of these studies scored below a critical threshold for low risk of bias. Prevalence of VR-related side effects was low (though adverse effects were rarely reported in studies).
Acute medical operations and surgery	Tas *et al.* (2022)	Meta-analysis	Children undergoing somatic medical procedures	14	Immersive forms of VR only	Found a moderate effect for VR exposure and distraction therapies (in separate analyses). These effects remained after removing low-quality studies and for sensitivity analysis of venous access and oncology procedures. Meta-regressions showed that interventions were not more efficacious for younger vs older children.
Acute medical operations and surgery	van der Linde-van den Bor *et al.* (2022)	Scoping Review	Adults and children undergoing surgery and/or radiation therapy	11	Education-/exposure-based methods	Identified a range of VR interventions that have been utilised, such as immersive videos, interactive games, 3D models and educational training systems. Significant reductions in preoperative anxiety were found in the majority of studies. Null effects were observed when VR was administered by a researcher, and not by a clinician. High levels of acceptance were found, though potential issues with generalisability, appropriateness, fidelity and realism were raised.
Acute medical operations and surgery	Wang *et al.* (2022b)	Narrative Synthesis	Adults undergoing an acute medical procedure	8	All relevant intervention types were included	Most of the included studies found positive effects, with patients generally perceiving that VR could improve anxiety outcomes. However, there were insufficient evidence in favour of VR over iPad distraction methods. While interventions were generally well tolerated and accepted, a lower acceptability of VR therapy appeared among older patients (who may show an increased anxiety about technology). Mild feelings of claustrophobia, discomfort, and cybersickness were also reported in some studies.
Acute medical operations and surgery	Yu *et al.* (2023)	Narrative Synthesis	Adults undergoing elective surgery	5	Education-/exposure-based methods	Studies of 'virtual operating room tour' (VORT) interventions showed mixed results for reducing pre- and post-operative anxiety. There was a lack of homogeneity in terms of the patients, surgery types, and measurement tools. The authors concluded that VORTs serve as a promising tool in alleviating operative anxiety in adults.

Acute medical operations and surgery	Zhang *et al.* (2023)	Scoping Review	Adults and children undergoing surgery	10	All relevant intervention types were included	XR provides a novel alternative to traditional information leaflets or conversations for minimising surgical anxiety. VR was the most studied form of technology, with most interventions taking place at the pre-operative (as opposed to intra-/post-operative) stage of care.
Cancer treatment and rehabilitation	Chow *et al.* (2021)	Narrative Synthesis	Adult and child oncology patients	6	Immersive forms of VR	Synthesis showed null changes in anxiety for patients undergoing medical procedures or chemotherapy. Analyses revealed a general trend toward reduced anxiety in VR. Methodological issues and small samples limited the strength of conclusions.
Cancer treatment and rehabilitation	Comparcini *et al.* (2023)	Narrative Synthesis	Children with haematological or solid cancers	8	Distraction-based methods	Found lower procedure-related anxiety scores in VR compared to control groups, both for self-reported and observer-reported measures. However, statistical significance was not reached in five of the eight included studies. The authors observed that the studies reporting patient benefits tended to use immersive VR systems.
Cancer treatment and rehabilitation	Rutkowski *et al.* (2021)	Meta-analysis	Adults undergoing chemotherapy	3	Distraction-based methods	Meta-analysis showed null differences between VR and control conditions in terms of reducing State Anxiety Inventory scores. Studies were too heterogeneous to be pooled, and there were low research standards in relation to sample sizes and study designs. Promising results have been shown in more recent articles, which employ modern scientific standards and advanced technology.
Cancer treatment and rehabilitation	Cheng *et al.* (2022)	Meta-analysis	Child oncology patients	5	Distraction-based methods	Meta-analysis showed large significant differences between VR and control groups. VR reduced anxiety about chemotherapy and needle-related treatments. High levels of heterogeneity were observed, but these were reduced by excluding low-quality studies.
Cancer treatment and rehabilitation	Czech *et al.* (2023)	Meta-analysis	Child oncology patients	4	Distraction-based methods	There was a large difference between the VR and control treatment conditions for reducing anxiety during standard oncologic care procedures. Despite reaching statistical significance, the included studies were too heterogeneous to be pooled.
Cancer treatment and rehabilitation	Grilo *et al.* (2023)	Narrative synthesis	Adults undergoing radiotherapy	7	Education-/exposure-based methods	Anxiety about radiotherapy decreased in almost all of the evaluated interventions. Similar changes were seen in some control groups. High heterogeneity of results emerged, which was attributed to varying levels of trait anxiety, treatment context, and VR exposure.
Dental procedures	Chen *et al.* (2021)	Meta-analysis	Adults with dental phobias	2	Education-/exposure-based methods (all immersive forms of VR)	Meta-analyses showed large, significant reductions in state and trait anxiety for dental phobias. These studies were based on a single session of VR therapy and small sample sizes. The authors argue that VR has emerged as a feasible tool for supporting the delivery of clinically-focused treatments and interventions.

Dental procedures	Cunningham *et al.* (2021)	Narrative Synthesis	Children undergoing dental examinations or treatment	3	All relevant intervention types were included	VR found to significantly reduce self-reported patient anxiety in the evaluated evidence. Noted that VR is mostly being used as a distraction tool, rather than for exposure (apart from in specialist clinical populations).
Dental procedures	Custodio *et al.* (2020)	Meta-analysis	Children aged 12 and below, undergoing dental treatment	2	Distraction-based methods	Found mixed evidence for VR as a distraction method for dental treatments. Observed significant differences for self-report anxiety when compared with control interventions. Evidence was evaluated as moderate in strength, due to imprecision of study measures.
Dental procedures	Lopez-Valverde *et al.* (2020)	Meta-analysis	Adults and children undergoing dental treatment	7	Distraction-based methods	Meta-analysis showed that paediatric dental anxiety significantly reduced in VR intervention groups. Positive effects were large and did not seem to be affected by publication bias. Lack of evidence obtained from adult populations and exposure-based methods.
Dental procedures	Martinez-Bernal *et al.* (2023)	Scoping Review	Adults and children undergoing oral cavity procedures	11	All relevant intervention types were included	The use of VR has grown exponentially in oral cavity procedures, with most studies employing passive, distraction-based methods. VR significantly decreased anxiety/fear in the majority of studies. Null effects were observed in a study of postoperative anxiety. Authors contend that patient acceptance may be lower in younger (vs older) children, who may have more difficulty using equipment.
Dental procedures	Yan *et al.* (2023)	Meta-analysis	Children receiving dental treatment	11	Distraction-based methods	Found a large effect for VR therapies reducing paediatric anxiety. Substantial heterogeneity was shown, due to differences in measures and treatment. 'Some concerns' or a 'high' risk of bias were appraised in all of the studies. Effects were larger for passive (vs active) VR, although there was a limited number of studies. No adverse events were reported in the included studies, however a small number of patients presented discomfort (in two studies).
Imaging procedures	Cataldo *et al.* (2023)	Scoping Review	Adults and children undergoing MRI scans	8	All relevant intervention types were included	Research data suggested that there is significant potential for VR reducing anxiety about scanning procedures. However, studies lacked consistency in their measurement tools and contained small sample sizes. The authors supported the future development of immersive, relatable, and easy to use VR applications.
Miscellaneous healthcare procedures	Addab *et al.* (2022)	Narrative Synthesis	Adults and children undergoing acute medical and/or dental procedures	32	Distraction-based methods	Synthesis supported the use of VR distraction methods for reducing procedural anxiety, although support was not as strong in adult (vs child) populations. Studies varied in their design, samples, VR protocol, and clinical context. Most studies used interactive games or immersive experiences within head-mounted VR devices.

Miscellaneous healthcare procedures	Eijlers *et al.* (2019)	Meta-analysis	Children undergoing a somatic medical procedure	7	All relevant intervention types were included	Found a large, robust effect for VR reducing anxiety in paediatric patients. Heterogeneity was high, due to outlying and low-quality studies (as well as different contexts). Meta-regressions suggested interventions were more efficacious for younger vs older children.
Miscellaneous healthcare procedures	Kilic *et al.* (2021)	Narrative Synthesis	Adults and children undergoing acute medical and/or dental procedures	15	All relevant intervention types were included	VR elicited positive effects on anxiety during dental, imaging, and needle-related procedures. Null effects observed for wound care. Most interventions were distraction-based, although benefits were also seen for exposure-based therapies. Heterogeneity was high, and methodological quality was appraised as relatively low.
Needle-related procedures	Czech *et al.* (2021)	Meta-analysis*	Children	2	Distraction-based methods	Data were too heterogeneous to be pooled for meta-analyses. Studies focused on markedly different clinical contexts and populations. A high risk of bias was appraised in the research, due to the allocation of groups based on gender and age characteristics.
Needle-related procedures	Gao *et al.* (2023)	Meta-analysis	Children	10	Distraction-based methods	VR interventions produced a moderate reduction in self-reported anxiety. There was significant heterogeneity observed, though effects remained whether compared to routine procedure or standard of care groups.
Needle-related procedures	Lluesma-Vidal *et al.* (2022)	Meta-analysis	Children	5	Distraction-based methods	VR produced a large reduction in perceptions of fear about needles, compared to various control conditions. Risk of bias was deemed high or unclear in most studies. VR was used as a distraction tool, typically during venipuncture/reservoir puncture procedures.
Needle-related procedures	Saliba *et al.* (2022)	Meta-analysis	Children	6	Distraction-based methods	Found a large reduction in patient anxiety after venous access procedures. High levels of heterogeneity were present, which were associated with the content of VR simulations (e.g., the use of cartoons vs natural scenery experiences).
Needle-related procedures	Wang *et al.* (2022a)	Meta-analysis	Children	6	Distraction-based methods	VR reduced needle-related anxiety in children and adolescents compared with control interventions. Large effects emerged and were robust for self-, parent-, nurse-, and physician-report measures. High levels of heterogeneity recorded in all analyses.
Wound care procedures	Fardin *et al.* (2020)	Meta-analysis	Adult burn patients	1	All relevant intervention types were included	Analysis of non-pharmacological interventions. Identified a study that used a VR-based range of motion game, where there was null evidence for significant reductions in anxiety (in comparison to routine care control conditions).

Wound care procedures	Smith *et al.* (2022)	Meta-analysis	Child burn patients	1	Distraction-based methods	Evaluated one study that examined situational anxiety during wound dressing removal procedures. Found a moderate effect in favour of VR, from a multi-modal distraction intervention.
Wound care procedures	Lan *et al.* (2023)	Meta-analysis	Child and adult burn patients	5	All relevant intervention types were included	Found that VR groups had significantly less anxiety than those in control groups during burn rehabilitation. Moderate effects were observed in the meta-analyses, though heterogeneity between trials were significant. Overall risk of bias was deemed low to moderate. Adherence rates to rehabilitation programmes were high in VR groups, and adverse events were generally low or minimal.
Wound care procedures	Scapin *et al.* (2018)	Narrative Synthesis	Child and adult burn patients	9	All relevant intervention types were included	VR reduces pain and its consequences, such as anxiety, during wound dressing and rehabilitation. Null findings were evident for some anxiety-related outcomes. Sensations of distraction and fun were suggested as possible mechanisms.

Reviews of General Health Anxiety Studies

Indication	Review Article	Review Type	Included Populations	No. of studies	Types of Interventions	Extracted Findings
Cancer treatment and rehabilitation	Ahmad *et al.* (2020)	Narrative Synthesis	Adult and child oncology patients	9	Distraction-based methods	VR generally proved effective in alleviating anxiety during painful treatment operations, though research is required in other clinical contexts. For adults, VR plus standard care was more effective than standard care alone during cancer treatments and hospitalisation.
Cancer treatment and rehabilitation	Bu *et al.* (2022)	Meta-analysis	Adults with breast cancer	4	All relevant intervention types were included	Noted a weak but consistent association between VR interventions and reductions in anxiety. Meta-analysis showed a large effect in favour of VR over standard education, pharmacological, or non-intervention controls. Substantial heterogeneity was observed, likely due to differences in intervention methods. Reports of cybersickness symptoms were generally infrequent but varied.
Cancer treatment and rehabilitation	Hao *et al.* (2023)	Meta-analysis	Adult and child oncology patients	3	All relevant intervention types were included	Within-group analysis showed that VR interventions significantly reduced self-reported anxiety in cancer patients undergoing rehabilitation. Large group differences emerged between VR and control groups. All anxiety studies were graded as 'good quality' and minimal VR-related adverse effects were reported overall.
Cancer treatment and rehabilitation	Leggiero *et al.* (2020)	Narrative Synthesis	Adult and child solid-tumour patients	8	All relevant intervention types were included	Most studies observed reduced anxiety following VR use, with positive patient experiences and few adverse effects reported. Studies were often underpowered (due to issues with attrition and study recruitment) and limited in methodological consistency.
Cancer treatment and rehabilitation	Obrero-Gaitan *et al.* (2022)	Meta-analysis	Adults with breast cancer	3	All relevant intervention types were included	VR therapies produced a large effect for reducing anxiety during post-surgery recovery, when compared with usual care. VR also proved more effective than smartphone apps. Improvements were linked to the 'distraction power' of VR and the increased activity permitted by these systems.
Cancer treatment and rehabilitation	Tian *et al.* (2022)	Meta-analysis	Adults with breast cancer	6	Immersive forms of VR	VR interventions elicited an overall positive effect on patient anxiety. Largest effects observed in studies using immersive and relaxing VR experiences, although physical training and education-based simulation tools were also implemented.
Cancer treatment and rehabilitation	Wu *et al.* (2023)	Meta-analysis	Adult and child oncology patients	7	All relevant intervention types were included	VR improved anxiety symptoms compared to control conditions. Large effects were reported with high levels of heterogeneity. Comparable effects emerged for distraction- and education-based methods, while improvements were observed in both child and adult patients. Adverse effects, such as cybersickness, were reported in a minority (17%) of studies and contributed to dropout.

Cancer treatment and rehabilitation	Zeng *et al.* (2019)	Meta-analysis	Adult oncology patients	4	All relevant intervention types were included	VR interventions had large, positive effects on reducing self-reported anxiety symptoms. Included studies were appraised to have a high risk of bias, except one case. Concerns related to a lack of randomization, power analysis, follow-up assessments and information about missing data/adverse effects.
Cancer treatment and rehabilitation	Zhang *et al.* (2022)	Meta-analysis	Adults with breast cancer	3	All relevant intervention types were included	VR interventions had a large significant effect on reducing anxiety, compared to control conditions. Heterogeneity proved considerably high in the pooled study data. Risk of bias was appraised as uncertain or high for all of the included trials.
Chronic Pain Management	Cortes-Perez *et al.* (2021)	Meta-analysis	Adults with Fibromyalgia Syndrome	3	All relevant intervention types were included	Found a medium effect in favour of VR relative to control interventions. Effects were observed without heterogeneity, though methods notably varied between trials. Studies were appraised as very low-quality, with a possible risk of publication bias.
Chronic Pain Management	Gava *et al.* (2022)	Meta-analysis	Adults with chronic pain conditions	2	All relevant intervention types were included	Meta-analyses found no superior effects of VR interventions when compared to control treatment groups and null differences between VR and exergame programmes. Analyses were based on anxiety data from physical therapies/exercise-based interventions.
Chronic Pain Management	Huang *et al.* (2022)	Meta-analysis	Adults and children with chronic pain conditions	10	All relevant intervention types were included	Participants in VR groups had lower levels of anxiety compared with control groups. A large pooled effect was observed, based on data from clinical and non-clinical studies. The authors concluded that VR could have greater potential for child (vs adult) patients.
Cardiac Rehabilitation	Bashir *et al.* (2023)	Meta-analysis	Adults with cardiovascular disease	4	All relevant intervention types were included	A significant improvement was found in VR groups compared to controls. These effects were small, but remained significant after removing non-immersive forms of VR. Risk of bias was judged to be high, or to have some concerns, for all of the included studies.
Cardiac Rehabilitation	Chen *et al.* (2022)	Meta-analysis	Adults with cardiovascular disease	3	All relevant intervention types were included	Found null, uncertain effects for VR reducing anxiety in patients undergoing cardiac rehabilitation. Analyses were mostly based on exercise- or education-based intervention data. Minimal levels of heterogeneity were observed.
Cardiac Rehabilitation	Turan-Kavradim *et al.* (2023)	Meta-analysis	Adults with cardiovascular disease	8	All relevant intervention types were included	Found a large, positive effect for VR interventions reducing patient anxiety. Studies showed significant heterogeneity, but very good stability. Evidence for the intervention effect were deemed low-level, based on Cochrane GRADE criteria.
Maternity	Baradwan *et al.* (2022)	Meta-analysis	Pregnant women	3	All relevant intervention types were included	VR significantly reduced anxiety scores during labour, based on simulating natural sceneries or showing images of newborns. Pooled effects were large, but highly heterogeneous. However, this heterogeneity could be minimised through the exclusion of a single study. Reports of adverse events were minimal during labour.

Maternity	Hajesmaeel-Gohari *et al.* (2021)	Scoping Review	Pregnant women	5	All relevant intervention types were included	The identified studies focused on various clinical settings, including curettage procedures, caesarean surgery, episiotomy repair, and general mother-foetus interactions. Most studies used VR headsets and reported decreases in anxiety, although 1 large-scale RCT showed null effects.
Maternity	Xu *et al.* (2022)	Meta-analysis	Pregnant women	7	All relevant intervention types were included	VR therapies reduced maternal anxiety during delivery. Large statistical effects were observed with high stability and heterogeneity. The authors noted that VR interventions were less effective in the context of pre-caesarean sections (when patient concerns vary and occur over longer timescales). The incidence of adverse events did not differ between control and VR groups.
