# Supplementary material for: Extended reality interventions for health and procedural anxiety: An overview of reviews
Source: Digit Health. 2026 Feb 11;12:20552076251411512. doi: 10.1177/20552076251411512 (PMC12901853; doi:10.1177/20552076251411512)
Supplement: sj-pdf-4-dhj-10.1177_20552076251411512 - Supplemental material for Extended reality interventions for health and procedural anxiety: An overview of reviews [file sj-pdf-4-dhj-10.1177_20552076251411512.pdf]

Supplementary File 4. Appraisal of included reviews based on AMSTAR-2 criteria.

Review Article	Q1	Q2	Q3	Q4	Q5	Q6	Q7	Q8	Q9	Q10	Q11	Q12	Q13	Q14	Q15	Q16	Overall
Ahmad *et al.* (2020)	Yes	No	Yes	PY	Yes	Yes	Yes	PY	PY	No	N/A*	N/A*	Yes	Yes	N/A*	No	Low
Baradwan *et al.* (2022)	Yes	No	No	PY	Yes	Yes	No	PY	Yes	No	Yes	Yes	Yes	Yes	Yes	Yes	Critically Low
Bashir *et al.* (2023)	Yes	Yes	No	PY	Yes	Yes	No	PY	Yes	No	Yes	No	Yes	Yes	Yes	Yes	Low
Bu *et al.* (2022)	Yes	Yes	No	PY	Yes	Yes	No	PY	Yes	No	No	No	Yes	Yes	No	Yes	Critically Low
Chen *et al.* (2021)	Yes	No	No	PY	No	Yes	No	PY	PY	No	Yes	No	No	Yes	Yes	Yes	Critically Low
Chen *et al.* (2022)	Yes	No	No	PY	Yes	Yes	No	PY	Yes	No	Yes	Yes	No	Yes	Yes	Yes	Critically Low
Chen et al. (2023)	Yes	No	No	PY	No	Yes	No	Yes	PY	No	Yes	Yes	Yes	No	Yes	Yes	Critically Low
Cheng *et al.* (2022)	Yes	Yes	No	PY	Yes	Yes	No	PY	Yes	No	Yes	Yes	Yes	Yes	Yes	Yes	Low
Chow *et al.* (2021)	Yes	Yes	No	No	Yes	No	Yes	PY	PY	No	N/A*	N/A*	Yes	Yes	N/A*	Yes	Low
Comparcini *et al.* (2023)	Yes	Yes	No	PY	Yes	Yes	Yes	PY	Yes	No	N/A*	N/A*	No	No	N/A*	No	Moderate
Cortes-Perez *et al.* (2021)	Yes	Yes	No	PY	Yes	Yes	No	PY	PY	No	Yes	Yes	Yes	Yes	Yes	Yes	Low
Cunningham *et al.* (2021)	Yes	Yes	No	PY	Yes	Yes	No	PY	Yes	No	N/A*	N/A*	No	Yes	N/A*	Yes	Critically Low
Custodio *et al.* (2020)	Yes	Yes	No	PY	Yes	No	Yes	PY	Yes	No	Yes	Yes	Yes	Yes	Yes	Yes	Moderate
Czech *et al.* (2021)	Yes	Yes	No	PY	Yes	No	Yes	PY	Yes	No	Yes	No	Yes	Yes	No	Yes	Low
Czech *et al.* (2023)	Yes	Yes	No	PY	Yes	Yes	Yes	PY	Yes	No	Yes	No	No	Yes	Yes	Yes	Low
Desmet *et al.* (2021)	Yes	No	No	No	No	No	No	PY	No	No	N/A*	N/A*	No	No	N/A*	Yes	Critically Low
Eijlers *et al.* (2019)	Yes	No	No	PY	Yes	Yes	No	PY	PY	No	No	Yes	Yes	Yes	Yes	Yes	Critically Low

Fardin *et al.* (2020)	Yes	No	No	PY	No	Yes	Yes	PY	Yes	No	Yes	No	Yes	Yes	No	Yes	Critically Low
Gaina *et al.* (2022)	Yes	No	No	PY	No	No	No	PY	No	No	N/A*	N/A*	No	No	N/A*	Yes	Critically Low
Gao *et al.* (2023)	Yes	Yes	No	PY	Yes	Yes	No	PY	Yes	No	Yes	No	No	Yes	Yes	Yes	Critically Low
Gava *et al.* (2022)	Yes	Yes	No	PY	Yes	Yes	No	PY	PY	No	Yes	No	Yes	Yes	Yes	Yes	Low
Grilo *et al.* (2023)	Yes	No	No	PY	Yes	Yes	No	PY	No	No	N/A*	N/A*	No	No	N/A*	Yes	Critically Low
Hao *et al.* (2023)	Yes	Yes	No	PY	Yes	Yes	No	PY	PY	No	Yes	Yes	Yes	Yes	Yes	Yes	Low
Huang *et al.* (2022)	Yes	Yes	No	PY	Yes	Yes	No	PY	Yes	No	Yes	No	No	Yes	Yes	Yes	Critically Low
Kilic *et al.* (2021)	Yes	Yes	No	PY	Yes	No	No	PY	Yes	No	N/A*	N/A*	Yes	Yes	N/A*	Yes	Low
Koo *et al.* (2020)	Yes	No	No	PY	Yes	Yes	No	No	Yes	No	Yes	Yes	Yes	Yes	Yes	Yes	Critically Low
Lan *et al.* (2023)	Yes	No	No	PY	Yes	Yes	No	PY	Yes	No	Yes	No	No	Yes	Yes	Yes	Critically Low
Leggiero *et al.* (2020)	Yes	No	Yes	PY	Yes	Yes	No	PY	No	No	N/A*	N/A*	No	Yes	N/A*	Yes	Critically Low
Lluesma-Vidal *et al.* (2022)	Yes	Yes	No	PY	Yes	Yes	No	PY	Yes	No	Yes	No	Yes	No	No	Yes	Critically Low
Lopez-Valverde *et al.* (2020)	Yes	No	No	PY	Yes	No	Yes	PY	Yes	No	Yes	No	Yes	Yes	Yes	Yes	Low
Obrero-Gaitan *et al.* (2022)	Yes	Yes	No	PY	Yes	Yes	No	PY	Yes	No	No	Yes	Yes	Yes	Yes	Yes	Critically Low
Rutkowski *et al.* (2021)	Yes	Yes	Yes	PY	Yes	Yes	No	PY	Yes	No	N/A*	N/A*	Yes	No	N/A*	Yes	Low
Saliba *et al.* (2022)	Yes	No	No	PY	No	No	No	PY	PY	No	Yes	Yes	Yes	Yes	No	Yes	Critically Low
Simonetti *et al.* (2022)	Yes	Yes	No	PY	Yes	Yes	No	Yes	Yes	No	Yes	Yes	Yes	Yes	Yes	Yes	Low
Smith *et al.* (2020)	Yes	No	No	PY	No	No	No	PY	PY	No	N/A*	N/A*	No	Yes	N/A*	Yes	Critically Low
Smith *et al.* (2022)	Yes	No	Yes	PY	Yes	No	No	PY	PY	No	Yes	Yes	No	Yes	No	Yes	Critically Low
Tas *et al.* (2022)	Yes	No	No	PY	Yes	Yes	No	PY	PY	No	Yes	Yes	Yes	Yes	Yes	Yes	Critically Low

Tian *et al.* (2022)	Yes	No	Yes	PY	Yes	Yes	No	PY	PY	No	No	No	Yes	No	No	Yes	Critically Low
Turan-Kavradim *et al.* (2023)	Yes	Yes	Yes	PY	Yes	Yes	No	PY	Yes	No	Yes	No	Yes	Yes	Yes	Yes	Low
Wang *et al.* (2022a)	Yes	No	No	PY	Yes	Yes	No	PY	Yes	No	Yes	Yes	Yes	Yes	No	Yes	Critically Low
Wu *et al.* (2023)	Yes	Yes	No	PY	Yes	Yes	No	PY	Yes	No	Yes	Yes	Yes	Yes	No	Yes	Critically Low
Xu *et al.* (2022)	Yes	Yes	No	PY	Yes	No	No	PY	Yes	No	Yes	Yes	Yes	Yes	No	Yes	Critically Low
Yan *et al.* (2023)	Yes	Yes	No	PY	Yes	Yes	No	PY	Yes	No	Yes	No	Yes	Yes	Yes	Yes	Low
Yu *et al.* (2023)	Yes	Yes	No	PY	Yes	Yes	No	Yes	Yes	No	N/A*	N/A*	No	Yes	N/A*	Yes	Critically Low
Zeng *et al.* (2019)	No	No	No	PY	No	No	No	PY	No	No	No	No	Yes	No	No	Yes	Critically Low
Zhang *et al.* (2022)	Yes	No	No	PY	Yes	Yes	No	PY	Yes	No	Yes	No	No	No	No	Yes	Critically Low

*Asterisks denote the reviews without meta-analysis, where specified criterion ratings were not applicable.
