# Supplementary material for: Extended reality interventions for health and procedural anxiety: An overview of reviews
Source: Digit Health. 2026 Feb 11;12:20552076251411512. doi: 10.1177/20552076251411512 (PMC12901853; doi:10.1177/20552076251411512)
Supplement: sj-pdf-5-dhj-10.1177_20552076251411512 - Supplemental material for Extended reality interventions for health and procedural anxiety: An overview of reviews [file sj-pdf-5-dhj-10.1177_20552076251411512.pdf]

Supplementary File 5. Summary of Extracted Meta-Analysis Data

Pooled effects reported for procedural anxiety studies

**Review Article**	**Indication**	**Studies**	**Metric**	**Effect Size**	**95% CI**	**I^2**
Chen *et al.* (2023)	Acute medical operations and surgery	5	SMD	-0.91	-1.43 to -0.39	86%
Koo *et al.* (2020)	Acute medical operations and surgery	10	SMD	-0.64	-1.08 to -0.20	90%
Simonetti *et al.* (2022)	Acute medical operations and surgery	6	PL	-0.34	-0.62 to -0.11	39%
Tas *et al.* (2022): VR exposure	Acute medical operations and surgery	4	SMD	-0.58	-1.15 to -0.01	87%
Tas *et al.* (2022): VR distraction	Acute medical operations and surgery	10	SMD	-0.74	-1.00 to -0.48	59%
Rutkowski *et al.* (2021)	Cancer treatment and rehabilitation	3	SMD	N/A*	N/A*	92%
Cheng *et al.* (2022)	Cancer treatment and rehabilitation	5	SMD	-1.47	-2.46 to -0.48	92%
Czech *et al.* (2023)	Cancer treatment and rehabilitation	4	SMD	-1.86	-2.98 to -0.73	93%
Chen *et al.* (2021)	Dental procedures (state anxiety data)	2	SMD	-1.44	-2.12 to -0.78	12%
Custodio *et al.* (2020)	Dental procedures	2	MD	-3.37	-4.57 to -2.18	71%
Lopez-Valverde *et al.* (2020)	Dental procedures	7	SMD	-1.75	-3.06 to -0.43	51%
Yan *et al.* (2023)	Dental procedures	11	SMD	-1.74	-2.46 to -1.02	95%
Eijlers *et al.* (2019)	Miscellaneous healthcare procedures	7	SMD	-1.32	-0.21 to -2.44	97%
Czech *et al.* (2021)	Needle-related procedures	2	SMD	N/A*	N/A*	93%
Gao *et al.* (2023)	Needle-related procedures	10	SMD	-0.61	-1.02 to -0.20	85%
Lluesma-Vidal *et al.* (2022)	Needle-related procedures	5	SMD	-1.26	-1.89 to -0.63	89%
Saliba *et al.* (2022)	Needle-related procedures	6	SMD	-0.89	-0.16 to -1.63	95%
Wang *et al.* (2022a)	Needle-related procedures	6	WMD	-2.79	-4.07 to -1.54	88%
Fardin *et al.* (2020)	Wound care procedures	1	SMD	-0.45	-1.3 to 0.4	N/A
Smith *et al.* (2022)	Wound care procedures	1	SMD	-0.58	-0.11 to -1.04	N/A
Lan *et al.* (2023)	Wound care procedures	5	SMD	-0.73	-1.35 to -0.11	67%

*studies were too heterogeneous to be pooled; CI: confidence interval; I^2: heterogeneity of the effect size estimate; MD: mean difference (non-adjusted); PL: Pooled Likelihood; SMD: standardized mean difference; WMD: weighted mean difference.

Pooled effects reported for general health anxiety studies

**Review Article**	**Indication**	**Studies (*k*)**	**Effect Metric**	**Effect Size**	**95% CI**	**I^2 (%)**
Bu *et al.* (2022)	Cancer treatment and rehabilitation	4	MD	-6.47	-7.21 to -5.73	83%
Hao *et al.* (2023)	Cancer treatment and rehabilitation	3	MD	-6.99	-9.73 to -4.25	77%
Obrero-Gaitan *et al.* (2022)	Cancer treatment and rehabilitation	3	SMD	-1.79	-2.70 to -0.91	NR
Tian *et al.* (2022)	Cancer treatment and rehabilitation	6	MD	-4.93	-8.00 to -1.87	92%
Wu *et al.* (2023)	Cancer treatment and rehabilitation	7	SMD	-0.83	-1.25 to -0.42	82%
Zeng *et al.* (2019)	Cancer treatment and rehabilitation	4	SMD	-3.03	-6.20 to 0.15	95%
Zhang *et al.* (2022)	Cancer treatment and rehabilitation	3	SMD	-2.07	-3.81 to -0.34	95%
Cortes-Perez *et al.* (2021)	Chronic Pain Management	3	SMD	-0.47	-0.91 to -0.03	0%
Gava *et al.* (2022)	Chronic Pain Management	2	SMD	0.03	-0.40 to 0.46	0%
Huang *et al.* (2022)	Chronic Pain Management	10	WMD	-1.30	-1.86 to -0.75	40%
Bashir *et al.* (2023)	Cardiac Rehabilitation	4	SMD	-0.32	-0.61 to 0.03	0%
Chen *et al.* (2022)	Cardiac Rehabilitation	3	SMD	-0.35	-0.70 to 0.01	0%
Turan-Kavradim *et al.* (2023)	Cardiac Rehabilitation	8	Hedge's g	-0.85	-1.55 to -0.14	92%
Baradwan *et al.* (2022)	Maternity	3	SMD	-1.15	-2.18 to -0.12	88%
Xu *et al.* (2022)	Maternity	7	SMD	-1.39	-1.99 to -0.78	89%

CI: confidence interval; I^2: heterogeneity of the effect size estimate; MD: mean difference (non-adjusted); NR: not reported; SMD: standardized mean difference; WMD: weighted mean difference.
