# Supplementary material for: Extended reality interventions for health and procedural anxiety: An overview of reviews
Source: Digit Health. 2026 Feb 11;12:20552076251411512. doi: 10.1177/20552076251411512 (PMC12901853; doi:10.1177/20552076251411512)
Supplement: sj-pdf-6-dhj-10.1177_20552076251411512 - Supplemental material for Extended reality interventions for health and procedural anxiety: An overview of reviews [file sj-pdf-6-dhj-10.1177_20552076251411512.pdf]

Supplementary File 6. Summary of Findings Table for implementation outcomes.

	**Distraction-based Intervention methods**	**Education- and Exposure-based methods**	**Combination of Intervention methods**	
**Acute medical operations and surgery**	***Paediatric groups****:* non-compliance rates for XR group close to 8%[39]	***Paediatric groups***: discomfort in some younger children[62], non-compliance rates were high (21%)[39] ***Combined groups***: high acceptance found, though potential issues with appropriateness and generalisability for users[41]; adverse effects rarely reported by patients[41,62]	***Paediatric groups****:* XR compliance was greater than or equal to controls[40] usability can be limited by inappropriate tools[42] ***Adults***: patients had a broadly positive perception of VR[70] ***Combined groups***: low prevalence of adverse effects[33,83]; high acceptability, though lower in older patients[33]	***Conclusions***: High levels of acceptability were found, though this is not the case for all patient groups or XR tools. Adverse effects rarely reported by patients or in studies
**Cancer treatment and rehabilitation**	***Paediatric groups****:* XR proved feasible and well-accepted[80] ***Combined groups***: adverse effects rarely reported in studies[36,84], infrequent cases of cybersickness[84]	***Combined groups***: adverse effects rarely reported by patients or in studies[84]	***Adults***: adverse effects rarely reported in studies[45,47], except some cybersickness cases[47,58] ***Combined groups***: minimal adverse effects[49], patients reported positive experiences/usability, though significant attrition evident[58]	***Conclusions***: Promising levels of acceptability and feasibility are found. Minimal adverse effects generally observed (except infrequent cases of cybersickness)
**Cardiac rehabilitation**	No identified review evidence	No identified review evidence	No synthesis of data relating to XR implementation	***Conclusions***: Lack of research evidence
**Chronic pain management**	No identified review evidence	No identified review evidence	***Adults***: ~10% of participants dropped out of XR intervention[44]	***Conclusions***: Lack of research evidence
**Dental procedures**	***Paediatric groups****:* minimal adverse effects reported in studies, although some users experienced discomfort[67]	No synthesis of data relating to XR implementation	***Paediatric groups***: lack of data on acceptability and adverse effects[35] ***Combined groups***: high satisfaction/acceptance, though limited in younger children[79]	***Conclusions***: High user acceptability shown, though research is needed in young children. Adverse effects rarely reported
**Imaging procedures**	No identified review evidence	No identified review evidence	***Combined groups***: high usability, though limited synthesis of data[53]	***Conclusions***: Lack of research evidence

**Maternity**	No identified review evidence	No identified review evidence	***Adults***: minimal adverse effects[86], sickness and dizziness experienced by some patients but at comparable rates to control interventions[88]	***Conclusions***: Lack of evidence for acceptability and usability. XR not associated with any additional adverse effects
**Needle-related procedures**	***Paediatric groups***: limited available data on patient acceptability[72]	No identified review evidence	No synthesis of data relating to XR implementation	***Conclusions***: Lack of research evidence
**Wound care procedures**	***Adults***: No adverse effects were reported by participants[81]	No identified review evidence	***Combined groups***: Minimal adverse effects generally reported by patients[37,38], though nausea experienced in a small number of cases[38]; adherence rates higher in XR than in controls[37]	***Conclusions***: Lack of evidence for acceptability and usability. Minimal adverse effects generally observed
	Conclusions: Lack of evidence around acceptability and usability. Minimal adverse effects were generally reported or observed	***Conclusions***: Lack of evidence around acceptability and usability. Minimal adverse effects were generally observed, though greater reporting of these events is necessary	***Conclusions***: High levels of acceptability and usability are shown, but research is needed in young children and older adults. Adverse effects rarely reported and are generally consigned to minor discomfort or cybersickness	
